# Robustness of Majorana modes to a disorder potential in atomic chains on a superconducting Rashba alloy

**DOI:** 10.1038/s41567-026-03322-3

**Published:** 2026-05-26

**Authors:** Harim Jang, Daniel Crawford, Khai That Ton, Lucas Schneider, Jens Wiebe, Makoto Shimizu, Harald O. Jeschke, Stephan Rachel, Roland Wiesendanger

**Affiliations:** 1 Department of Physics, University of Hamburg, Hamburg, Germany; 2 Department of Physics and Nanoscience Center, University of Jyväskylä, Jyväskylä, Finland; 3 Department of Physics, Graduate School of Science, Kyoto University, Kyoto, Japan; 4 Research Inst. for Interdisciplinary Science, Okayama University, Okayama, Japan; 5 School of Physics, University of Melbourne, Parkville, Victoria Australia

**Keywords:** Topological matter, Surfaces, interfaces and thin films

## Abstract

Majorana modes offer great potential for fault-tolerant quantum computation due to their topological protection. However, intrinsic disorder makes the unambiguous detection of Majorana modes difficult in the commonly used hybrid superconductor–semiconductor nanowire platform. By contrast, for magnet–superconductor hybrid systems, Majorana modes were theoretically predicted to be very robust against disorder, but no experimental confirmation of this has been reported so far. Here we demonstrate that these modes are indeed robust in one-dimensional spin chains constructed from individual iron atoms on a Rashba surface alloy with proximity-induced superconductivity. Although the chains exhibit perfect crystalline order, we observe nanoscale potential disorder of the BiAg_2_/Ag(111)/Nb(110) heterostructure by scanning tunnelling microscopy. However, this does not prevent the emergence of zero-energy modes at both ends of the atomic chains, in agreement with tight-binding calculations showing that these modes are only found in the topologically non-trivial regime of the phase diagram. Our results explain the earlier observation of zero-energy Majorana modes in disordered Fe chains on other superconducting substrates, and may provide an avenue for the realization of Majorana qubits.

## Abstract (web summary)

The presence of Majorana zero modes is often difficult to confirm experimentally due to the detrimental effects of disorder. Now, a chain of magnetic atoms on a proximatized surface is shown to exhibit robust Majorana states with disorder present.

## Main

Atomic magnetic chains on superconducting (SC) substrates have been proposed as a promising platform for the observation of topological superconductivity and associated zero-energy Majorana modes^1–7^. Signatures for Majorana modes have indeed been found experimentally for self-assembled Fe chains on an SC Pb(110) substrate by detecting zero-energy peaks in the differential tunnelling conductance (d*I*/d*V*) spectra measured by scanning tunnelling spectroscopy (STS) at the ends of such Fe chains^8–10^. However, a topological gap could not be detected for this particular magnet–superconductor hybrid (MSH) system even down to a temperature of 30 mK. Moreover, the influence of disorder^11,12^, as present for these self-assembled Fe chains on Pb(110), could not be clarified in these early investigations. Subsequent STS studies on disorder-free atom-by-atom-constructed close-packed Fe chains on SC Re(0001) confirmed the emergence of zero-energy states at both ends of perfectly ordered chains as a function of chain length, whereas a topological gap could not be detected at the measurement temperature of 300 mK (refs. ^13,14^). Further artificially designed MSH platforms (for example, on the basis of diluted spin chains^15^, quantum spin chains^16^ or SC alloy substrates^17^) have been investigated. Recently, bottom-up-fabricated Mn chains on ultimately clean SC Nb(110) substrates allowed the experimental observation of a spin–orbit-coupling-induced gap as large as 180 μeV in one of the multiorbital Shiba bands^18^ and chain-length-dependent oscillations of the low-energy modes simultaneously probed by STS at both ends of disorder-free atomic chains^19^. These hybridization-induced splitting oscillations have been theoretically identified early on as a smoking gun for the experimental confirmation of the elusive Majorana modes^20,21^.

In this work, we present an exciting model-type MSH system consisting of atom-by-atom-constructed Fe chains of various lengths on a BiAg_2_/Ag(111) surface alloy with proximity-induced superconductivity from a Nb(110) substrate. The BiAg_2_ surface alloy grown epitaxially on a single-crystalline Ag(111) thin film is known to exhibit a large Rashba-type spin–orbit coupling^22–24^, which is expected to favour a large topological gap of the MSH system comprising an SC Nb(110) substrate, that is, the elemental superconductor with the highest SC transition temperature of 9.3 K. We show that the long-range nature of the Yu–Shiba–Rusinov (YSR) states of Fe atoms residing on hollow sites with respect to the BiAg_2_ lattice allows for considerable hybridization between the Fe atoms even for a spacing of two atomic lattice sites. On the basis of low-temperature STS measurements of the Fe chains on the SC BiAg_2_/Ag(111)/Nb(110) substrate as a function of chain length, the emergence of highly localized zero-energy modes at both ends of these perfectly crystalline Fe chains can be directly observed in real space. Tight-binding calculations suggest that this MSH system resides in a topologically non-trivial regime and that the experimentally observed zero-energy end states can be associated with Majorana modes. Interestingly, the BiAg_2_/Ag(111)/Nb(110) substrate is found to exhibit potential disorder, in contrast to earlier experiments involving SC single-crystalline Re(0001) and Nb(110) substrates. The potential disorder of the BiAg_2_/Ag(111)/Nb(110) substrate, as revealed by atomic-resolution scanning tunnelling microscopy (STM) measurements, affects the spatial distribution of the finite-energy YSR bulk states, but does not prevent the observation of zero-energy end states, in agreement with model calculations taking a similar potential disorder distribution, as observed experimentally, into account. Our results provide direct proof for the robustness of Majorana modes in MSH systems even in the presence of disorder^25,26^.

## Tailored artificial hybrid materials design

**Fig. 1:**
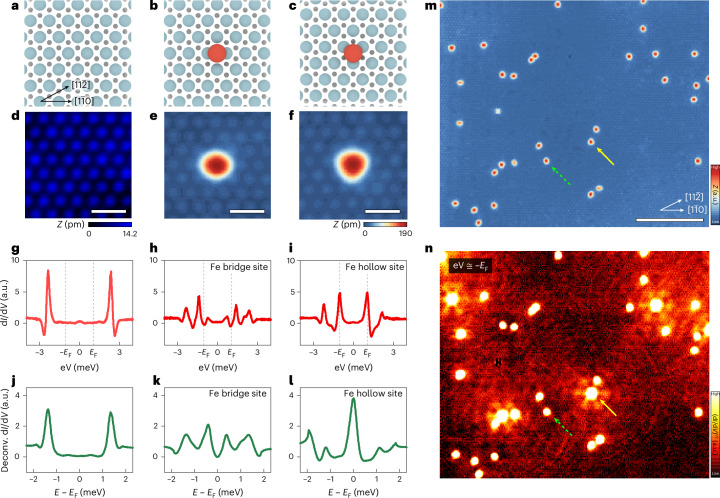
YSR states of single Fe adatoms on the BiAg_2_ surface alloy on Ag(111)/Nb(110). **a**–**c**, Top views of the schematic of the surface structures of the (√3 × √3) BiAg_2_/Ag(111)*R*30° surface alloy without adatom (**a**) and with single Fe adatom on the bridge (**b**) and hollow site (**c**). The large red spheres at the centre, the pale blue and small dark sphere symbolize Fe adatoms, Bi atoms and Ag atoms, respectively. The crystallographic directions are given in **a**. **d**–**f**, Atomically resolved constant-current STM images of the bare BiAg_2_ surface alloy with a Bi lattice period of 485 pm along the [$1\bar{1}0$] direction (**d**), the Fe bridge-site adatom (**e**) and the Fe hollow-site adatom (**f**). Scale bars, 1 nm. **g**–**i**, d*I*/d*V* spectra as a function of energy (eV) measured on the BiAg_2_ surface alloy (**g**) and on top of the Fe bridge-site adatoms (**h**) and Fe hollow-site adatoms (**i**). The vertical dashed lines correspond to the sample’s *E*_F_, which is determined based on the SC gap of the Nb tip. **j**–**l**, Numerically deconvoluted d*I*/d*V* spectra for the BiAg_2_ surface alloy (**j**) and Fe bridge-site adatoms (**k**) and Fe hollow-site adatoms (**l**). **m**, Constant-current STM image of the BiAg_2_/Ag(111)/Nb(110) surface with Fe bridge-site (green dashed arrow) and Fe hollow-site (yellow arrow) adatoms. Scale bar, 10 nm. **n**, Spectroscopic d*I*/d*V* map obtained for eV = −*E*_F_. The YSR states of the Fe bridge-site adatoms appear highly localized on the atomic scale, whereas the YSR state of the Fe hollow-site adatoms exhibits long-range spatial oscillations extending over more than 10 nm. Measurement parameters: *T* = 4.2 K, *V*_mod_ = 40 μV and *V*_stab_ = 5 mV; *I*_stab_ = 0.4 nA (**d**–**f**), *I*_stab_ = 1 nA (**g**–**i**) and *I*_stab_ = 0.4 nA (**m** and **n**). Source data

To combine a large spin–orbit coupling with *s*-wave superconductivity, as an important ingredient for achieving a topological SC state, we have designed and realized a special type of heterostructure consisting of an SC Nb(110) substrate, epitaxially grown Ag(111) islands with proximity-induced superconductivity^27,28^, and a monolayer of a BiAg_2_(111) surface alloy on top exhibiting a √3 × √3*R*30° superstructure (Fig. 1a,d). The d*I*/d*V* measurements performed at *T* = 4.2 K on top of the BiAg_2_ surface alloy clearly reveal the characteristics of an induced SC state with an energy gap *Δ*_S_ of 1.31 meV (Fig. 1g). Since an SC Nb tip was used to enhance the energy resolution, the Fermi level (*E*_F_) value of the sample is shifted by the size of the SC energy gap *Δ*_T_ of the Nb tip (Fig. 1g, dashed line). A numerically deconvoluted spectrum is presented in Fig. 1j (Supplementary Section 1 provides details of the deconvolution procedure). Individual Fe atoms were subsequently deposited on the cold BiAg_2_/Ag(111)/Nb(110) substrate, thereby preventing surface diffusion. Atomic-resolution STM measurements reveal two distinct adsorption sites for single Fe adatoms (Fig. 1e,f), namely, bridge and hollow sites, for which the Fe adatoms are coordinated by two or three Bi atoms, respectively (Fig. 1b,c).

The d*I*/d*V* spectra measured above individual Fe adatoms on the SC BiAg_2_/Ag(111)/Nb(110) substrate exhibit discrete bound states inside the SC gap, the so-called YSR states (Fig. 1h,i shows the as-measured spectra and Fig. 1k,l shows the deconvoluted spectra). For the Fe bridge-site adatom, there are clearly visible peaks at *E* − *E*_F_ = ±0.40 meV related to a pair of YSR in-gap states, whereas the spectroscopic features at *E* − *E*_F_ = ±1.3 meV are caused by the SC coherence peaks. By contrast, the tunnelling spectrum of the Fe hollow-site adatom exhibits a pronounced YSR state located at *E*_F_.

Besides the strong adsorption-site dependence of the YSR bound-state energies, there is also a marked difference in the spatial extension of YSR states for Fe adatoms on bridge and hollow sites. Figure 1m shows a large-scale constant-current STM image of the BiAg_2_/Ag(111)/Nb(110) surface with several Fe adatoms adsorbed on either bridge or hollow sites (examples are indicated by a dashed green arrow and a yellow arrow, respectively). Interestingly, the corresponding spectroscopic d*I*/d*V* map obtained for eV = −*E*_F_ (Fig. 1n) reveals that the pronounced YSR state of hollow-site Fe adatoms exhibits long-range oscillations over more than 10 nm with a periodicity of around 2 nm (Fig. 1n, yellow arrow), whereas the bridge-site Fe adatoms show states spatially localized to atomic-scale dimensions (Fig. 1n, green arrow). The amplitude of the long-range YSR state oscillations decays inversely proportional to the distance from the hollow-site Fe adatoms, suggesting that the YSR bound states originate from an exchange coupling to an effectively two-dimensional (2D) superconductor associated with the BiAg_2_ monolayer film (Supplementary Section 2).

On the basis of our atomic-scale spectroscopic studies of the site-specific YSR states, we can conclude that the hollow-site Fe adatoms are the most promising as elemental building blocks for the construction of spin chains on BiAg_2_/Ag(111)/Nb(110) in view of achieving a topologically non-trivial SC state: first, the YSR state energy is already close to *E*_F_, making it a promising candidate for engineering YSR bands crossing *E*_F_. Second, the YSR state of the hollow-site Fe adatoms is of the long-range nature, thereby facilitating the hybridization between YSR states of Fe adatoms even at nanometre-scale distances and, therefore, the formation of YSR bands.

## Emergence of zero-energy end states in bottom-up-constructed Fe chains

**Fig. 2:**
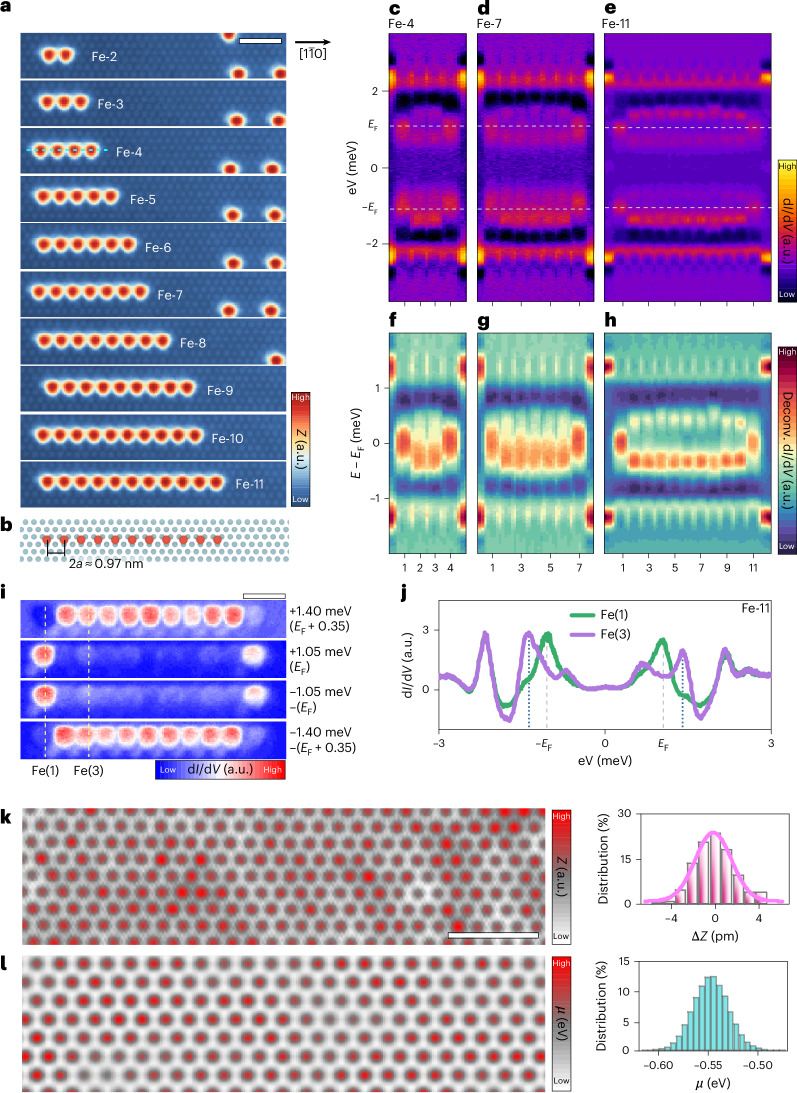
Bottom-up-fabricated Fe chains on SC BiAg_2_/Ag(111)/Nb(110) and their spectral characteristics. **a**, Constant-current STM images of artificially constructed Fe-*n* chains from Fe-2 (top) to Fe-11 (bottom) on the BiAg_2_ surface alloy, where *n* is the number of Fe hollow-site atoms in the chain. All the distances between Fe hollow-site atoms are twice the Bi–Bi distance: 2*a* ≈ 0.97 nm. **b**, Schematic of the Fe-11 chain on BiAg_2_. **c**–**e**, d*I*/d*V* line profiles of Fe chains along the centre of the chains’ axis (horizontal dashed line in **a** as an example) for the representative Fe chains Fe-4 (**c**), Fe-7 (**d**) and Fe-11 (**e**), as measured with an SC Nb tip. The horizontal dashed lines correspond to the sample’s *E*_F_ determined from the SC gap of the Nb tip. For **c**–**h**, the *x* axis denotes the sequentially numbered Fe atom from the left side of the chains. **f**–**h**, Numerically deconvoluted d*I*/d*V* line profiles from **c**–**e** (Supplementary Section 1). **i**, d*I*/d*V* maps taken on the Fe-11 chain at representative energies. The vertical dashed lines correspond to the positions of the first and third Fe atoms from the left side of the chain. **j**, Comparison between the d*I*/d*V*(eV) spectrum for the edge Fe atom Fe(1) and the Fe(3) site. The vertical dashed and dotted lines correspond to the sample’s *E*_F_ and eV = ±1.40 meV, respectively, that is, the energies chosen for the d*I*/d*V* maps in **i**. **k**, Atomically resolved constant-current STM image of the BiAg_2_ surface alloy on Ag(111)/Nb(110), showing nanoscale spatial variations in the LDOS. A histogram of the measured *Z*-height values above the Bi-atom sites is presented in the panel on the right, along with a Gaussian curve fit. Scale bars, 2 nm (**a**, **i** and **k**). **l**, Theoretical model of the disordered BiAg_2_ surface involving a correlated potential disorder *µ*_*i*_ to the substrate sites. Although the tight-binding model only involves Bi sites, to aid the eye, we additionally use a cubic interpolation to plot the substrate potential between Bi sites. A histogram of the Gaussian disorder is shown to the right. Measurement parameters: *T* = 4.2 K, *V*_stab_ = 5 mV, *V*_mod_ = 40 μV; *I*_stab_ = 0.2 nA (**a**), *I*_stab_ = 0.5 nA (**c** and **d**); *I*_stab_ = 1 nA (**e**, **i** and **j**); *I*_stab_ = 0.4 nA (**k**). Note that a different STM tip apex was used for **c** and **d** compared with **e**, **i** and **j**, where the latter features a slightly enhanced energy resolution. Source data

We crafted well-defined spin chains by individual Fe-atom manipulation to hollow sites, linearly arranged along the [$1\bar{1}0$] direction with respect to the BiAg_2_(111) lattice, and with a given interatomic spacing of 2*a* ≈ 0.97 nm. Topographic STM snapshots of the systematic construction of Fe chains with a variable length of two (Fe-2) up to eleven (Fe-11) atoms are shown in Fig. 2a. By using spin-polarized STM, we confirmed a ferromagnetically ordered state of such atomic Fe chains, in agreement with ab initio calculations (Supplementary Sections 3 and 9).

Energy-dependent d*I*/d*V* line profiles were obtained along all Fe-*n* chains, and some representative as-measured as well as numerically deconvoluted datasets are presented in Fig. 2c–h, respectively (Supplementary Section 4 shows the evolution of the spectroscopic d*I*/d*V* line profiles for all Fe-*n* chains). For Fe-*n* chains with *n* > 2, pronounced tunnelling conductance peaks at *E*_F_ (as measured with the SC Nb tip), that is, zero-energy edge states in the deconvoluted spectra, were found at both ends of the chains, with a linewidth of about 0.2 meV (Supplementary Section 4). It is worth noting that the spatially highly localized zero-energy edge states emerging in Fe-*n* chains (*n* > 2) have a fundamentally different origin compared with the YSR state of an individual hollow-site Fe adatom being located at *E*_F_, because the sizable hybridization between YSR states of neighbouring Fe adatoms lifts the degeneracy of the YSR bound-state energies for Fe-2 in 2*a* and even 3*a* chains (Supplementary Section 5). The strong localization of zero-energy modes at the ends of relatively short chains was discussed and explained previously^29^. Inside the chains, the YSR band develops at around ±0.35 meV, resulting from the hybridization between the YSR states of neighbouring Fe hollow-site adatoms.

Figure 2i presents the spectroscopic d*I*/d*V* maps of the Fe-11 chain at energies of ±*E*_F_ and ±(*E*_F_ + 0.35 meV). Zero-energy edge states clearly show up by considerable spectral weight only for the Fe hollow-site adatoms at the chain ends and only at *E*_F_. On the other hand, the d*I*/d*V* intensity at both ends is highly suppressed at finite energies of the YSR bands, which only show up inside the chain. A direct comparison between the as-measured tunnelling spectra of edge (Fe(1)) and middle atoms (Fe(3)) of the Fe-11 chain is presented in Fig. 2j (these positions are highlighted by vertical dashed lines in Fig. 2i; all of the as-measured d*I*/d*V*(*V*) data and the deconvoluted spectra of each Fe adatom within the Fe-11 chain are presented in Supplementary Section 6).

## Intrinsic disorder in the BiAg_2_ surface potential

When examining the spectroscopic d*I*/d*V* maps of the Fe-11 chain at finite energies (Fig. 2i, top and bottom) in more detail, one can recognize a spatial variation in the d*I*/d*V* intensity from one Fe atom to the next. This is associated with a local variation in the YSR state energies and contributes to the observed width of the YSR band (Fig. 2f–h). We have carefully investigated the origin of this effect and found that it is connected with a nanoscale surface potential disorder typical for the BiAg_2_ surface alloy prepared on Ag(111)/Nb(110) and not found on bare surfaces of elemental superconductors, such as Re(0001) (refs. ^14,15^) or Nb(110) (refs. ^18,19^). Even though the Bi atoms form a well-ordered hexagonal lattice (Fig. 1d), a spatial variation in the local density of states (LDOS) on the nanoscale can clearly be recognized in large-scale constant-current STM images (Fig. 2k). The observed nanoscale electronic inhomogeneity most probably originates from local strain effects in the bulk of Ag(111) nanostructures grown on Nb(110) (ref. ^30^) or from structural inhomogeneities at the Ag(111)/Nb(110) interface. The degree of disorder can be quantified by the distribution of recorded relative *Z*-height values above the Bi-atom sites, where stronger disorder increases the width of the distribution (Fig. 2k, right). Despite the presence of an intrinsic potential disorder on the BiAg_2_ surface, which affects the spatial distribution of the finite-energy YSR bulk states, it does not influence the spatial homogeneity of the proximity-induced SC state of the BiAg_2_ surface alloy (Supplementary Section 7) and it does not prevent the observation of the zero-energy end states of the atomic Fe chains on the SC BiAg_2_/Ag(111)/Nb(110) heterostructure (Fig. 2i and Supplementary Section 8).

## Robustness of Majorana zero modes

**Fig. 3:**
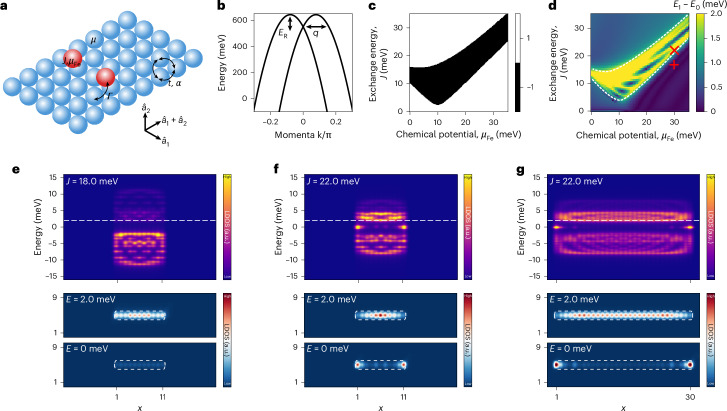
Theoretical modelling of Fe chains on SC BiAg_2_/Ag(111)/Nb(110). **a**, Illustration of a triangular net formed by Bi atoms (blue), with Fe adatoms on hollow sites (red). The model parameters are indicated. Superconductivity is applied only to the Bi atoms. **b**, Fit of the spin-split substrate bands to previous experiments on the BiAg_2_ surface alloy^31^. **c**, Topological phase diagram for an infinite chain as a function of *µ*_Fe_ and *J* (*Γ* = 35 meV), with the black region being topologically non-trivial. **d**, Topological phase diagram for a finite chain (*L* = 11) with the adatoms placed on the hollow sites of a much larger substrate; the white dashed lines surround regions in a parameter space with *E* = 0 ground state. The colour map indicates the energy gap between the ground state and the first excited state. The red cross and plus symbols correspond to parameter pairs (*µ*_Fe_, *J*) for a topological and trivial regime, respectively, used in **e**–**g**. **e**–**g**, LDOS(*E*, *x*) plots for the trivial phase (*J* = 18 meV, *µ*_Fe_ = 30 meV) (**e**), topological phase for *L* = 11 (*J* = 22 meV, *µ*_Fe_ = 30 meV) (**f**) and topological phase for *L* = 30 (*J* = 22 meV, *µ*_Fe_ = 30 meV) (**g**). The bottom panels show the LDOS(*x*, *y*) plots for *E* = 0, and for the first excited state, as indicated by the white dashed lines in the panels above.

To support the interpretation of the experimental results, we constructed a minimal tight-binding model involving superconductivity to analyse atomic Fe chains on BiAg_2_/Ag(111)/Nb(110) (Supplementary Section 10). To this end, we only considered the top-surface Bi atoms forming a hexagonal lattice with period *a* as the substrate, where Fe atoms are placed on the hollow sites of the Bi lattice with distance 2*a* between neighbouring Fe atoms (Fig. 3a). The Bi atoms are coupled to an isotropic SC *s*-wave order parameter *Δ*_0_, whereas the Fe atoms have a magnetic moment of size *J*. Thus, our model mimics the SC proximity effect, when we observe (topological) superconductivity on the Fe chain. Despite being minimal, the model’s parameter space is far too large; our strategy here was to use experimental insights and results from ab initio calculations, to reduce this parameter space as much as possible and in the most realistic way. For the normal state, we fitted a parabolic, spin-split substrate band with standard Rashba spin–orbit coupling to the quasiparticle interference measurements of ref. ^31^ (Fig. 3b and Supplementary Section 10). This allows us to determine reasonable parameters for the hopping, chemical potential and Rashba spin–orbit coupling (*t*, *µ*, *α*) = (100, –545, 25) meV of the substrate, by matching the Rashba energy *E*_R_ and scattering vector *q*. Although *Δ*_Nb_ = 1.51 meV, and up to 87% of that was measured on the BiAg_2_ surface owing to a strong SC proximity effect, we must use a substantially larger value of *Δ*_0_ for our purely 2D model to account for the absence of a three-dimensional SC substrate. Although, in the experiment, the proximity-induced SC gap measured on the chain *Δ* is similar to the bulk gap *Δ*_0_, in our model, the absence of a three-dimensional SC substrate results in *Δ* < *Δ*_0_. We chose *Δ*_0_ = 70 meV and a value for the Fe–Bi hybridization of *Γ* = 35 meV to match the experimentally observed spectral features in Fig. 2j, corresponding to an upper limit of the minigap of *Δ*_mini_/*Δ* ≈ 0.05/1.5, estimated from the experimental energy resolution, and a YSR bandwidth of approximately *Δ*/4 (Fig. 2e,h). Next, we performed density functional theory calculations (Supplementary Section 9) for 2*a*-Fe chains on BiAg_2_/Ag(111)/Nb(110), which turned out to be ferromagnetically coupled (approximately −1.3 meV for unit moments) with energetically preferred out-of-plane moments, in line with the spin-polarized STM measurements (Supplementary Section 3). The remaining parameters are the chemical potential of Fe atoms, *µ*_Fe_, and the effective magnetic moment *J*. To guarantee that the energy of the single YSR state of a hollow-site Fe adatom assumed in the model is *E*_YSR_ ≈ 0, as observed experimentally (Fig. 1i,l), we have to satisfy the relation *µ* ≈ *J* + *Γ*/3. The resulting one-dimensional model is in topological class D (refs. ^32,33^) with a *Z*_2_ invariant. This is readily verified by computing the *Z*_2_ invariant for an infinitely long chain (periodic boundary conditions), leading to the phase diagram shown in Fig. 3c. Next, we focused on chain length *L* = 11 on a substrate with 400 × 10 Bi atoms and open-boundary conditions. Such large substrates are necessary to observe the SC coherence peaks of the substrate at *E* ≈ *Δ*_0_. In Fig. 3d, we show a typical phase diagram depending on *J* and *µ*_Fe_, featuring a sizable region of topological superconductivity. The topologically non-trivial regime is identified by (1) states at *E* = 0 (Fig. 3d, white dashed line) and (2) a finite gap *E*_1_ − *E*_0_ (Fig. 3d, colour plot). The cross (plus) symbols in Fig. 3d correspond to parameters guaranteeing topological (trivial) superconductivity and *E*_YSR_ ≈ 0 of a single impurity on a hollow site.

We considered three cases: (1) an *L* = 11 chain with *J* = 18 meV (Fig. 3d, plus symbol), being topologically trivial with an induced minigap *Δ*_triv_ = 2 meV; (2) an *L* = 11 chain with *J* = 22 meV (Fig. 3d, cross symbol), being topologically non-trivial with an induced minigap *Δ*_topo_ = 2 meV; (3) we also show an example for a longer chain (*L* = 30 chain, *J* = 22 meV, topological regime, induced minigap *Δ*_topo_ = 2 meV) to demonstrate that our results are robust on varying the system size (*μ* = 30 meV for all three cases). We show the LDOS(*E*, *x*) along the chain for these three cases in Fig. 3e–g, along with the corresponding LDOS(*x*, *y*) for *E* = 0 close to the chain, and LDOS(*x*, *y*) at the energy of the band gap. In both LDOS(*E*, *x*) and LDOS(*x*, *y*) at *E* = 0, localized Majorana modes are indicative of the topological phase and can be clearly distinguished from the bulk states. In the trivial phase, there are no zero-energy end states.

**Fig. 4:**
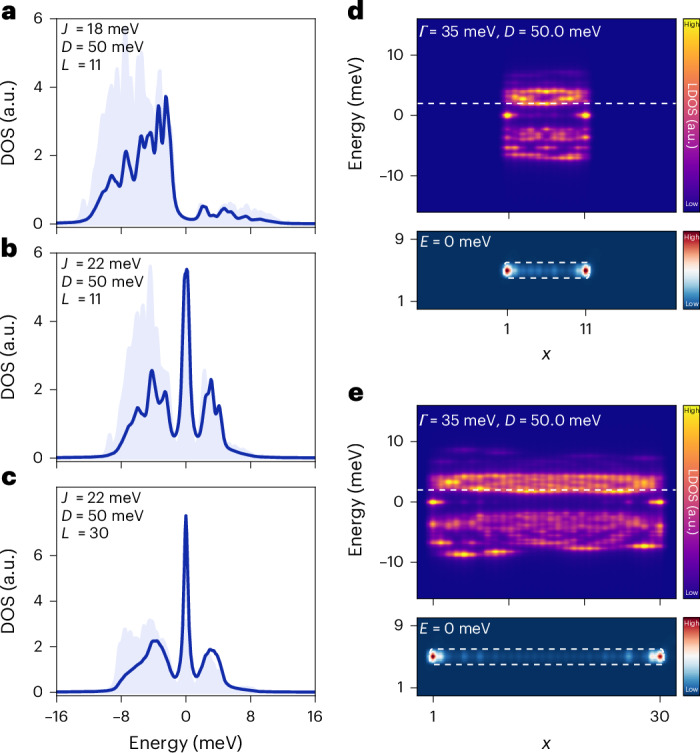
Theoretical analysis of the influence of substrate potential disorder on Majorana zero modes. **a**–**c**, Disorder-averaged DOS at the ends of Fe chains in the trivial phase with *L* = 11 (**a**), topological phase with *L* = 11 (**b**) and topological phase with *L* = 30 (**c**). The solid lines indicate the mean and the shaded regions indicate twice the standard deviation for 100 disorder realizations. **d**,**e**, LDOS(*E*, *x*) plots for two disorder realizations in the topological phase for chain lengths of *L* = 11 (**d**) and *L* = 30 (**e**). The white dashed lines show the minigap edge, in the corresponding clean limit. The bottom panels show the LDOS(*x*, *y*) plots at *E* = 0. For all plots, we used *µ*_Fe_ = 30 meV; in **d** and **e**, we used *J* = 22 meV. Source data

We now turn to the disorder analysis of the BiAg_2_ surface alloy. We modelled the disordered BiAg_2_ surface by applying a correlated potential disorder *µ*_*i*_ to the substrate sites (Fig. 2l). We used a Gaussian noise source similar to the experimental data (Fig. 2k), and applied a low-pass filter (Supplementary Section 11) to eliminate short-wavelength correlations. We chose a cut-off frequency such that there are nanoscale correlations in the disorder distribution (Fig. 2l), similar to the experiment (Fig. 2k). We selected the strength *D* of the disorder distribution such that the hard minigap just closes and only a soft minigap remains; at the same time, the Majorana modes remain well defined and are clearly visible within the LDOS(*E*, *x*) plots (Fig. 4d,e). Furthermore, we chose the width of the Gaussian source *D* such that the resulting LDOS is similar to experiment, up to *D* ≈ *Δ*. We computed 100 disorder realizations based on the three cases discussed above. We plot the disorder-averaged density of states (DOS; Fig. 4a–c) taken at one chain end (the DOS at the other end is essentially identical after disorder averaging), highlighting the effects of disorder on the YSR band and the end states. Although disorder can localize the zero-energy states, we did not find spurious zero-energy end states in the trivial phase in any of our simulations. By contrast, the zero-bias peak in the DOS due to the Majorana modes is robust to disorder over an extended range of disorder strengths. Due to the spin-triplet correlations, in all cases, disorder results in a soft minigap (Supplementary Section 10). We also show LDOS(*E*, *x*) in the topological phase for a single but representative disorder realization (Fig. 4d,e), for *L* = 11 and *L* = 30 chains. In both cases, the edge of the soft gap has shifted to a lower energy due to disorder. Although the Majorana modes remain localized, in both cases, there is an asymmetry in the spectral weight of the Majorana modes, reflecting the substrate disorder. Similarly, the bulk states are not periodic due to the disordered substrate. All results remain qualitatively unchanged when considering other types of disorder (Supplementary Section 11), thereby demonstrating the universal behaviour and robustness of Majorana modes in disordered MSH structures. Our findings may offer an explanation why Majorana modes could be observed earlier at the ends of highly disordered Fe nanowires self-assembled on SC Pb(110) substrates^8–10^. Moreover, on the basis of our results, MSH systems may provide a superior platform for the demonstration of robust Majorana qubits required for fault-tolerant topological quantum computation^34–36^.

## Methods

### Sample and STM tip preparation

The single-crystal Nb(110) surface was prepared by several high-temperature flashes up to a temperature of *T* ≈ 1,800 K. Extended Ag(111) islands were epitaxially grown on the oxygen-reconstructed Nb(110) surface via a multiple-step process. First, two monolayers of Ag were deposited as a wetting layer on Nb(110) at *T* ≈ 600 K. As the second step, the formation of nuclei of Ag islands on the Ag wetting layer was achieved at a lower temperature of *T* ≈ 400 K. More Ag was then deposited at an elevated temperature of *T* ≈ 650 K, leading to Ag(111) island growth around the nuclei. Islands with a thickness of 10–30 nm were obtained. As the last step, the substrate was held at an elevated temperature for a few minutes to reduce lattice defects and disorder. Subsequently, 0.33 monolayer of Bi atoms were deposited on the Ag(111) surface at *T* ≈ 160 K, followed by post-annealing, leading to a well-ordered BiAg_2_ surface alloy. Finally, Fe adatoms were deposited on the BiAg_2_ surface and keeping the substrate temperature below 7 K. All of the Fe chains were constructed in the bottom-up manner by using STM-based lateral atom manipulation techniques at tunnelling gap resistances of *R*_T_ ≈ 30–200 kΩ. A mechanically cut polycrystalline Nb wire was used for the STM/STS measurements. The Nb-tip apexes were shaped by touching the sample surface gently until high energy and spatial resolutions were obtained. Spin-polarized YSR tips were prepared by picking up a few Fe adatoms from the surface, forming a Fe cluster at the SC tip apex^37^.

### STM/STS measurements

The experiments were performed with a commercial SPECS STM system^38^ (Fig. 1m,n and Supplementary Figs. 2 and 6) and a custom-built STM operated in a single-shot He-3 cryostat connected to an ultrahigh-vacuum chamber system^39^. Constant-current STM images were obtained by stabilizing the tunnelling current *I*_stab_ across the superconductor(Nb)–insulator (vacuum)–superconductor (BiAg_2_/Ag/Nb) junction via the proportional–integral feedback loop and applying a fixed bias voltage *V*_stab_. For measurements of the d*I*/d*V* spectra as a function of energy (eV), the feedback loop was open and the d*I*/d*V* values were recorded by sweeping the sample bias voltage *V*. The d*I*/d*V* line profiles were also measured identically, but the junction was newly stabilized at each point. A standard lock-in technique was used with a small modulation voltage *V*_mod_ (r.m.s) of frequency *f*_mod_ on top of the applied d.c. bias voltage *V*. The range of *f*_mod_ was from 1.2 kHz to 4.5 kHz. The d*I*/d*V* maps were acquired by using the constant-contour method, that is, by first measuring the surface corrugation (*Z*-height profile) at a fixed bias voltage *V*_stab_ and afterwards measuring once again with a different bias *V*, but following the surface contour as determined before.

## Online content

Any methods, additional references, Nature Portfolio reporting summaries, source data, extended data, supplementary information, acknowledgements, peer review information; details of author contributions and competing interests; and statements of data and code availability are available at 10.1038/s41567-026-03322-3.

## Supplementary information

Supplementary Information Supplementary Sections 1–11, Figs. 1–11 and references.

## Source data

Source Data Fig. 1 Source data for Fig. 1.

Source Data Fig. 2 Source data for Fig. 2.

Source Data Fig. 4 Source data for Fig. 4.

## Data Availability

The datasets generated during this study are available from the corresponding authors on reasonable request. Source data are provided with this paper.

## References

[1] Martin, I. & Morpurgo, A. F. Majorana fermions in superconducting helical magnets. *Phys. Rev. B* **85**, 144505 (2012).

[2] Nadj-Perge, S., Drozdov, I. K., Bernevig, B. A. & Yazdani, A. Proposal for realizing Majorana fermions in chains of magnetic atoms on a superconductor. *Phys. Rev. B* **88**, 020407 (2013).

[3] Braunecker, B. & Simon, P. Interplay between classical magnetic moments and superconductivity in quantum one-dimensional conductors: toward a self-sustained topological Majorana phase. *Phys. Rev. Lett.* **111**, 147202 (2013). 24138267 10.1103/PhysRevLett.111.147202

[4] Pientka, F., Glazman, L. I. & von Oppen, F. Topological superconducting phase in helical Shiba chains. *Phys. Rev. B* **88**, 155420 (2013).

[5] Klinovaja, J., Stano, P., Yazdani, A. & Loss, D. Topological superconductivity and Majorana fermions in RKKY systems. *Phys. Rev. Lett.* **111**, 186805 (2013). 24237550 10.1103/PhysRevLett.111.186805

[6] Kim, Y., Cheng, M., Bauer, B., Lutchyn, R. M. & Das Sarma, S. Helical order in one-dimensional magnetic atom chains and possible emergence of Majorana bound states. *Phys. Rev. B* **90**, 060401(R) (2014).

[7] Lo Conte, R., Wiebe, J., Rachel, S., Morr, D. K. & Wiesendanger, R. Magnet-superconductor hybrid quantum systems: a materials platform for topological superconductivity. *Riv. Nuovo Cimento* **47**, 453–545 (2024).

[8] Feldman, B. E. et al. High-resolution studies of the Majorana atomic chain platform. *Nat. Phys.* **13**, 286–291 (2017).

[9] Nadj-Perge, S. et al. Observation of Majorana fermions in ferromagnetic atomic chains on a superconductor. *Science* **346**, 602–607 (2014). 25278507 10.1126/science.1259327

[10] Jeon, S. et al. Distinguishing a Majorana zero mode using spin-resolved measurements. *Science* **358**, 772–776 (2017). 29025997 10.1126/science.aan3670

[11] Ahn, S., Pan, H., Woods, B., Stanescu, T. D. & Das Sarma, S. Estimating disorder and its adverse effects in semiconductor Majorana nanowires. *Phys. Rev. Mater.* **5**, 124602 (2021).

[12] Woods, B. D., Das Sarma, S. & Stanescu, T. D. Charge-impurity effects in hybrid Majorana nanowires. *Phys. Rev. Appl.* **16**, 054053 (2021).

[13] Kim, H. et al. Toward tailoring Majorana bound states in artificially constructed magnetic atom chains on elemental superconductors. *Sci. Adv.* **4**, eaar5251 (2018). 29756034 10.1126/sciadv.aar5251 PMC5947976

[14] Rachel, S. & Wiesendanger, R. Majorana quasiparticles in atomic spin chains on superconductors. *Phys. Rep.* **1099**, 1–28 (2025).

[15] Küster, F. et al. Non-Majorana modes in diluted spin chains proximitized to a superconductor. *Proc. Natl Acad. Sci. USA* **119**, e2210589119 (2022). 36215505 10.1073/pnas.2210589119 PMC9586262

[16] Liebhaber, E. et al. Quantum spins and hybridization in artificially-constructed chains of magnetic adatoms on a superconductor. *Nat. Commun.* **13**, 2160 (2022). 35443753 10.1038/s41467-022-29879-0 PMC9021194

[17] Mier, C. et al. Atomic manipulation of in-gap states in the β-Bi_2_Pd superconductor. *Phys. Rev. B* **104**, 045406 (2021).

[18] Schneider, L. et al. Topological Shiba bands in artificial spin chains on superconductors. *Nat. Phys.* **17**, 943–948 (2021).

[19] Schneider, L. et al. Precursors of Majorana modes and their length-dependent energy oscillations probed at both ends of atomic Shiba chains. *Nat. Nanotechnol.* **17**, 384–389 (2022). 35256768 10.1038/s41565-022-01078-4 PMC9018407

[20] Das Sarma, S., Sau, J. D. & Stanescu, T. D. Splitting of the zero-bias conductance peak as smoking gun evidence for the existence of the Majorana mode in a superconductor-semiconductor nanowire. *Phys. Rev. B* **86**, 220506 (2012).

[21] Stanescu, T. D., Lutchyn, R. M. & Das Sarma, S. Dimensional crossover in spin-orbit-coupled semiconductor nanowires with induced superconducting pairing. *Phys. Rev. B* **87**, 094518 (2013).

[22] Ast, C. R. et al. Giant spin splitting through surface alloying. *Phys. Rev. Lett.* **98**, 186807 (2007). 17501597 10.1103/PhysRevLett.98.186807

[23] Bihlmayer, G., Blügel, S. & Chulkov, E. V. Enhanced Rashba spin-orbit splitting in Bi/Ag(111) and Pb/Ag(111) surface alloys from first principles. *Phys. Rev. B* **75**, 195414 (2007).

[24] El-Kareh, L., Sessi, P., Bathon, T. & Bode, M. Quantum interference mapping of Rashba-split Bloch states in Bi/Ag(111). *Phys. Rev. Lett.* **110**, 176803 (2013). 23679756 10.1103/PhysRevLett.110.176803

[25] Awoga, O. A., Björnson, K. & Black-Schaffer, A. M. Disorder robustness and protection of Majorana bound states in ferromagnetic chains on conventional superconductors. *Phys. Rev. B* **95**, 184511 (2017).

[26] Peeters, C., Hodge, T., Mascot, E. & Rachel, S. Effect of impurities and disorder on the braiding dynamics of Majorana zero modes. *Phys. Rev. B* **110**, 214506 (2024).

[27] Schneider, L. et al. Proximity superconductivity in atom-by-atom crafted quantum dots. *Nature* **621**, 60–65 (2023). 37587348 10.1038/s41586-023-06312-0 PMC10482682

[28] Schneider, L. et al. Scanning tunneling spectroscopy study of proximity superconductivity in finite-size quantized surface states. *Phys. Rev. B* **110**, L100505 (2024).

[29] Peng, Y., Pientka, F., Glazman, L. I. & von Oppen, F. Strong localization of Majorana end states in chains of magnetic adatoms. *Phys. Rev. Lett.* **114**, 106801 (2015). 25815952 10.1103/PhysRevLett.114.106801

[30] Tomanic, T. et al. Local-strain mapping on Ag(111) islands on Nb(110). *Appl. Phys. Lett.* **101**, 063111 (2012).

[31] Schirone, S. et al. Spin-flip and element-sensitive electron scattering in the BiAg_2_ surface alloy. *Phys. Rev. Lett.* **114**, 166801 (2015). 25955067 10.1103/PhysRevLett.114.166801

[32] Ryu, S., Schnyder, A. P., Furusaki, A. & Ludwig, A. W. W. Topological insulators and superconductors: tenfold way and dimensional hierarchy. *New J. Phys.* **12**, 065010 (2010).

[33] Kitaev, A. Periodic table for topological insulators and superconductors. *AIP Conf. Proc.* **1134**, 22–30 (2009).

[34] Kitaev, A. Y. Fault-tolerant quantum computation by anyons. *Ann. Phys.* **303**, 2–30 (2003).

[35] Nayak, C., Simon, S. H., Stern, A., Freedman, M. & Das Sarma, S. Non-Abelian anyons and topological quantum computation. *Rev. Mod. Phys.* **80**, 1083–1159 (2008).

[36] Alicea, J., Oreg, Y., Refael, G., von Oppen, F. & Fisher, M. P. A. Non-Abelian statistics and topological quantum information processing in 1D wire networks. *Nat. Phys.* **7**, 412–417 (2011).

[37] Schneider, L., Beck, P., Wiebe, J. & Wiesendanger, R. Atomic-scale spin-polarization maps using functionalized superconducting probes. *Sci. Adv.* **7**, eabd7302 (2021). 33523927 10.1126/sciadv.abd7302 PMC7817096

[38] Löptien, P., Zhou, L., Khajetoorians, A. A., Wiebe, J. & Wiesendanger, R. Superconductivity of lanthanum revisited: enhanced critical temperature in the clean limit. *J. Phys. Condens. Matter* **26**, 425703 (2014). 25272968 10.1088/0953-8984/26/42/425703

[39] Wiebe, J. et al. A 300 mK ultra-high vacuum scanning tunneling microscope for spin-resolved spectroscopy at high energy resolution. *Rev. Sci. Instrum.* **75**, 4871–4879 (2004).

