# Supplementary material for: Robustness of Majorana modes to a disorder potential in atomic chains on a superconducting Rashba alloy
Source: Nat Phys. 2026 May 26;22(7):1050–6. doi: 10.1038/s41567-026-03322-3 (PMC13423794; doi:10.1038/s41567-026-03322-3)
Supplement: Supplementary file 1 — Supplementary Sections 1–11, Figs. 1–11 and references. [file 41567_2026_3322_MOESM1_ESM.pdf]

nature physics

Supplementary information https://doi.org/10.1038/s41567-026-03322-3

Robustness of Majorana modes to a disorder potential in atomic chains on a superconducting Rashba alloy

In the format provided by the authors and unedited

Supplementary Information

Table of contents

Section 1. Numerical deconvolution of tunneling spectra from Superconductor-Insulator-Superconductor (SIS) junctions

Section 2. Long-range nature of YSR states of single Fe adatoms on the $BiAg_2$ surface alloy

Section 3. Ferromagnetic state of the bottom-up fabricated 2*a*-Fe chains on the $BiAg_2$ surface alloy

Section 4. Evolution of the spectral characteristics of 2*a*-Fe chains as a function of chain length

Section 5. Hybridization induced splitting of the YSR state for an Fe adatom pair

Section 6. As-measured dI/dV(V) and the deconvoluted spectra of each Fe adatom in the Fe-11 chain

Section 7. Spatial homogeneity of proximity-induced superconductivity in the $BiAg_2$ surface alloy

Section 8. Comparison of spectral characteristics of Fe-11 chains at various levels of disorder

Section 9. Ab initio calculations

Section 10. Tight binding calculations: Minimal model and phase diagram

Section 11. Analysis of correlated disorder

References (*40*-*59*)

Supplementary Figs. S1 to S11

Section 1. Numerical deconvolution of tunneling spectra from Superconductor-Insulator-Superconductor (SIS) junctions

In scanning tunneling spectroscopy (STS), the use of superconducting (SC) Nb-tips offers the advantage of an enhanced energy resolution in differential tunneling conductance (d*I*/d*V*) spectra[40]. On the other hand, it prevents the direct measurement of the surface local density of states (LDOS) due to the convolution with the strongly energy-dependent LDOS of the SC Nb-tip. A numerical deconvolution procedure of the tunneling spectra was adopted to derive the surface LDOS from the as-measured d*I*/d*V*(*V*) spectra. The convoluted d*I*/d*V* spectrum can be expressed as follows:

$$\frac{dI}{dV}(V,T) \propto \int dE \left(\frac{\partial}{\partial V} \rho_{\mathrm{T}}(E+eV)\left[f(E+eV,T) - f(E,T) \right] + \rho_{\mathrm{T}}(E+eV) \frac{\partial}{\partial V} f(E+eV,T) \right) \rho_{\mathrm{S}}(E) \tag{S1}$$

where ρ_{T}, ρ_{S}, f, T, and V represent the tip LDOS, surface LDOS, Fermi function, temperature, and bias voltage, respectively. The following isotropic s-wave Bardeen-Cooper-Schrieffer (BCS) form of the DOS along with a finite quasiparticle lifetime (Γ), a so-called Dynes DOS, was taken into account for describing the effect of the Nb-tip with SC energy gap Δ_{T} [41,42]:

$$\rho_{\mathrm{T}}(E,\Delta_{\mathrm{T}},\Gamma) = \mathrm{Re}\left[\frac{E - i\Gamma}{\sqrt{(E - i\Gamma)^2 - \Delta_{\mathrm{T}}^2}} \right] \tag{S2}$$

Mathematical approaches and the detailed steps to find a proper solution of the unknown surface LDOS function, ρ_{S}, can be found elsewhere[43]. A key procedure used in this study involves a matrix approximation to the integral form and treating the measured raw spectrum as a summation of the surface LDOS with a finite level of error (ε) that includes an adjustable parameter. By introducing a $N \times N$ matrix **A** (which is known), equation (S1) can be rewritten in a discrete form:

$$\frac{dI}{dV}(V_j,T)+\varepsilon_j \propto \sum_i^N A_{i,j}(V_i,T)\cdot \rho_{\mathrm{T},i}(V_i)$$
$$A_{i,j}=\frac{\partial}{\partial V}\rho_{\mathrm{T}}(E_j+eV_i)\left[f(E+eV_i,T)-f(E_j,T)\right]+\rho_{\mathrm{T}}(E_j+eV_i)\frac{\partial}{\partial V}f(E_j+eV_i,T) \quad \text{(S3)}$$

A condition for the solution with a minimized error can be obtained with the following relation

$$\left[\rho_{\mathrm{S}}(V)\right]\cong\left(\mathbf{A}+\gamma\mathbf{B}\right)^{-1}\cdot\left[\frac{dI}{dV}(V)\right] \quad \text{(S4)}$$

The elements of the $N \times N$ matrix **B** are defined by using the elements of the inverse of the matrix **A**: ($\mathbf{A}^{-1}$) = $\alpha_{i,j}$ and ($\mathrm{B}_{l,k}$) = $\alpha_{k-2,l} - 4\alpha_{k-1,l} + 6\alpha_{k,l} - 4\alpha_{k+1,l} + \alpha_{k+2,l}$, where $\alpha_{-2,l} = -\alpha_{0,l}$, $\alpha_{-1,l} = \alpha_{N+1,l} = 0$, and $\alpha_{N+2,l} = \alpha_{N,l}$. Note that both ρ_{S} and the measured dI/d$V(V)$ are written in vector form. Since the positive constant γ determines the degree of the oscillatory error in the deconvoluted spectra, it plays an important factor in the deconvolution process: higher γ yields a smoother result. The size of the matrix N was chosen between 500 to 800, and the error correction term γ was determined for each spectrum by iterating the numerical deconvolution until the oscillatory features in the resulting spectrum were suppressed. It is worth noting that the introduction of a finite value Γ is necessary for the deconvolution process, otherwise the tip DOS is not smooth enough to apply this mathematical approach. Even though Γ cannot be obtained from the experiment, it was roughly chosen in the range of 1% - 2% of Δ_{T}, a typical value for BCS superconductors for temperatures far below the SC transition temperature. The leftover leading term to resolve the surface LDOS is the tip gap Δ_{T}.

There are two different ways of determining the SC energy gap of the Nb-tip. The first one utilizes the sub-gap structure of an asymmetric SIS Josephson junction in the dI/d$V(V)$ spectrum[44]. Asymmetric SIS junctions with different SC energy gaps of the two SC electrodes, Δ_{T} and Δ_{S}, can often be found in our experimental set-up of Nb-vacuum-$BiAg_2$/Ag/Nb junctions, where the superconductivity of $BiAg_2$ is induced by the proximity effect from the bulk Nb substrate below. Fig. S1 shows an exemplary dI/d$V(V)$ spectrum with a sub-gap structure at two close tip-sample distances, where Δ_{S} and Δ_{T} are more pronounced due to a lower junction resistance. The small peak at zero-bias in the blue spectrum is a signature of the Josephson supercurrent and the peak near 0.35 mV

is a mirrored virtual state of Δ_S with respect to the Δ_T via thermal excitation. Thus, determining Δ_T (and Δ_S) from the sub-gap structure in the dI/dV(V) spectrum is straightforward, which is indicated by a dashed line at positive sample bias voltage. Once the Δ_T for a specific tip apex has been determined properly, the Δ_S at arbitrary positions can be extracted from the fact that the main coherence peaks in the dI/dV(V) spectrum are showing up at energies being the sum of both SC energy gaps of tip and surface.

The second way of Fermi level (E_F) determination is based on utilizing the thermally-mirrored in-gap states of the dI/dV(V) spectrum obtained on top of an Fe adatom. Even if the tip-sample distance is too large to observe sub-gap features of an asymmetric SIS junction, thermal excitations of YSR states result in mirrored peaks with respect to Δ_T, as shown in Fig. S1B. The values of the SC energy gaps determined by both procedures were found to be in good agreement.

Section 2. Long-range nature of YSR states of single Fe adatoms on the $BiAg_2$ surface alloy

The YSR state, i.e. a quasiparticle excitation resulting from the exchange coupling between a magnetic impurity and Cooper pairs, of a single magnetic adatom decays as a function of distance in the SC substrate[45-47]. In the case of a host superconductor that has a three-dimensional (3D) electronic structure, the LDOS of the YSR state is highly localized near the magnetic atom and decays inversely proportional to the square of the distance, where the typical length scale is on the order of a few Fermi wavelengths[48-50]. Reducing the effective dimensionality of the host superconductor leads to a much weaker decay, thus the YSR states extend far beyond the length scale given by the Fermi wavelength, as reported for a few SC platforms with (quasi) two-dimensional (2D) electronic structure such as Fe atoms in 2H-$NbSe_2$ or in La(0001)[50,51]. The spatial decay of the LDOS of a YSR state for the case of an effective 2D system is inversely proportional to the distance from the center of the adatom, and its detailed spatial pattern depends on the shape of the Fermi surface. STM has been used to reveal the long-range YSR states and their Fermi surface related properties more than tens of nanometers away from the center of the magnetic impurity[50-52].

Fig. S2 shows the spatial extension of YSR states of single Fe adatoms on the SC surface alloy of $BiAg_2$ on Ag(111)/Nb(110). In Fig. S2A, a representative constant-current STM image of a surface area exhibiting both Fe bridge-site (green arrow) and Fe hollow-site (yellow arrow) adatoms is presented. In Figs. S2B and S2C, the prototypical $\mathrm{d}I/\mathrm{d}V(eV)$ spectra probed on the Fe hollow- and bridge-site adatoms, respectively, are shown together with the spectrum of the $BiAg_2$ surface for comparison. Vertical lines indicate energies for which corresponding $\mathrm{d}I/\mathrm{d}V$ maps are shown in Figs. S2D-I (see Methods for $\mathrm{d}I/\mathrm{d}V$ map measurements). These $\mathrm{d}I/\mathrm{d}V$ maps of the area shown in Fig. S2A reveal the long-range nature of the YSR state of Fe hollow-site adatoms over more than ten nanometers. In contrast, all states of Fe bridge-site adatoms are highly localized within atomic distances. The oscillatory patterns of the long-range YSR state of Fe hollow-site adatoms are anisotropic and their detailed shape depends on the energy. Regardless of the shape, a strong amplitude of the YSR state of Fe hollow-site adatoms is observed along the $[1\bar{1}0]$ direction.

The spatial modulation and its anti-phasing between the particle-like (positive bias) and hole-like (negative bias) YSR state components are clearly visible in the $\mathrm{d}I/\mathrm{d}V$ maps. They are highlighted by the line cuts of the $\mathrm{d}I/\mathrm{d}V$ maps near a Fe hollow-site adatom, as shown in Fig. S2J-L, plotted on a semi-logarithmic scale. A sinusoidal spatial modulation with a period of around 1.85 nm is

observed at $E{-}E_F = \pm 0.2$ meV (Fig. S2K) and at $E{-}E_F = \pm 0.66$ meV (Fig. S2L), where the phase difference between the particle- and hole-like excitations is close to π. In contrast, we found no or only a small phase difference for the YSR state at the Fermi level (Figs. S2J), because the particle- and hole-like components of this YSR state overlap and are not energetically resolved. The upper envelope of the spatially decaying states of Fe hollow-site adatoms follows an $[\exp(-Ax)]/x$ dependence (dashed lines in Figs. S2J-L) for all energies, where A is a constant related with the phase differences and SC coherence length, indicating that they are coupled to an effectively 2D superconductor, presumably realized by the proximitized $BiAg_2$ surface alloy. On the other hand, all YSR states of Fe bridge-site adatoms are highly localized within atomic distances, as can be seen from the line cuts of the d*I*/d*V* maps near the Fe bridge-site adatom (see Fig. S2M-O). In this case, the upper envelope of the spatially decaying states follows an $[\exp(-Ax)]/x^2$ dependence for all energies (dashed lines in Figs. S2M-O)). This indicates a coupling to an effectively 3D superconductor, presumably realized by the underlying Ag layer[50].

Section 3. Ferromagnetic state of the bottom-up fabricated 2*a*-Fe chains on the $BiAg_2$ surface alloy

The spin texture of the bottom-up fabricated Fe chains on $BiAg_2$/Ag(111)/Nb(110) has been investigated by spin-polarized STM (SP-STM)[53] in the presence of an external magnetic field. A spin-sensitive tip was prepared by picking up a few magnetic Fe atoms from the surface until it showed clear signatures of YSR states in the d*I*/d*V* spectrum[37]. Fig. S3A (left) shows a schematic view of SP-STM measurements performed with a tip exhibiting a YSR-state (YSR-tip). The superconductivity of the $BiAg_2$ surface alloy induced by the bulk Nb(110) substrate was entirely quenched by applying an external magnetic field of 0.4 T, while the SC Nb-tip still remained in a SC state. Fig. S3A (right) shows the expected d*I*/d*V* spectrum of an Fe adatom within the chain, where the relative intensities of both components of the YSR state (indicated by arrows) vary depending on the spin polarizations of the YSR state at the tip and of the Fe adatom. It is worth noting that the two peaks arising from the YSR state on the tip typically exhibit an asymmetry in intensity (dashed line). The changes in intensity of the YSR peaks are opposite for the two different cases of parallel (red solid line) and antiparallel (blue solid line) spin configurations of tip and surface adatom. The spin asymmetry *A* is defined by the relative change in YSR peak intensities normalized to the intrinsic YSR peak intensity of the tip[37]:

$$A = \frac{a_e/a_{e,0} - a_h/a_{h,0}}{a_e/a_{e,0} + a_h/a_{h,0}} \qquad \text{(S5)}$$

where a_e and a_h represent the peak intensities of the electron- and hole-like YSR components as measured on the Fe adatom within the chain, and $a_{e,0}$ and $a_{h,0}$ represent the measured ones on the bare $BiAg_2$ surface, respectively. Figs. S3B and S3C show schematic views (top) and line profiles of *A* (open symbols) for a ferromagnetically and antiferromagnetically ordered spin chain made up of nine adatoms, respectively. The value of *A* on a non-magnetic substrate has to be zero by definition, indicated by dashed lines in each panel. All *A* values have the same sign for the ferromagnetically ordered spin chain (Fig. S3B), whereas the sign is expected to alternate for the antiferromagnetically ordered spin chain (Fig. S3C).

A constant-current STM image of a representative Fe chain is displayed in Fig. S3D. A spectroscopic d*I*/d*V*(*V*) line profile along this Fe chain (horizontal dashed line in Fig. S3D) is presented

in Fig. S3E, while individual dI/dV(eV) spectra of four of the Fe adatoms starting from the left side of the chain in Fig. S3D are displayed in Fig. S3F, together with a dI/dV(eV) spectrum measured above the $BiAg_2$/Ag(111)/Nb(110) substrate for comparison. The red solid lines represent fits with two Lorentzian functions to these spectra from which the spin asymmetry A of the YSR peak intensities has been extracted. Fig. S3G shows the line profile of the determined spin asymmetry A along the Fe chain. It is in agreement with the expected line profile for a ferromagnetically ordered spin chain (Fig. S3B), but in clear disagreement with the assumption of an antiferromagnetic spin chain (Fig. S3C).

Section 4. Evolution of the spectral characteristics of 2*a*-Fe chains as a function of chain length

Fig. S4 shows the evolution of the spectral characteristics of the Fe-*n* chains with increasing chain length up to $n = 11$, where n is the number of Fe hollow-site adatoms within the respective chain. The d*I*/d*V*(*eV*) line profiles were measured along the Fe chains (i.e. in $[1\bar{1}0]$ direction on the $BiAg_2$ surface), together with two substrate d*I*/d*V* spectra outside the chains for comparison. All Fe-(*n*-1) chains were extended to Fe-*n* chains by placing an additional Fe hollow-site adatom next to the right end of the chain. The spectral characteristics of the Fe-*n* ($n > 2$) chains remain qualitatively the same with increasing chain length: zero-energy edge states at both ends of the chain and finite energy YSR bands inside the chain.

The observed zero-energy end states exhibit a line-width of about 0.2 meV, as determined from a Lorentzian function fit (see red line on Fe(1) in Fig. S5). Several contributions to this measured line-width exist: the intrinsic width of the zero-energy end state, instrumental broadening as well as residual thermal broadening effects (though a SC tip has been used to minimize the latter contribution).

The spectral features at around $E–E_F = \pm 0.35$ meV are a signature of YSR band formation. The width of the YSR bands is about 0.2 meV, as determined from Lorentzian function fits (see red line on Fe(3) in Fig. S5). However, we note that the details of the in-gap band structure cannot be captured from the experimental observation due to the narrow YSR band width and the spatial inhomogeneities of the YSR band resulting from potential disorder (see Fig. S9). This is in contrast to previously studied magnetic chains bottom-up fabricated on SC Nb(110) substrates, revealing unprecedented insights into the details of YSR band formation[18,19,54].

Section 5. Hybridization induced splitting of the YSR state for an Fe adatom pair

Fig. S6 shows the effect of hybridization upon the formation of a pair of hollow-site Fe adatoms with a distance of $3a$ ~ 1.49 nm on the SC $BiAg_2$ surface alloy. Spectroscopic $\mathrm{d}I/\mathrm{d}V(eV)$ line profiles were measured for an individual Fe hollow-site adatom as well as for a pair of such Fe adatoms with a spacing of $3a$ (along the $[1\bar{1}0]$ direction relative to the $BiAg_2$ surface) and afterwards deconvoluted according to the procedure outlined in section S1.

The $\mathrm{d}I/\mathrm{d}V(eV)$ spectrum of the individual Fe hollow-site adatom clearly shows a single spectroscopic feature at the Fermi level, consistent with the spectroscopic data presented in Fig. 1L of the main text. When a second Fe adatom is placed at a distance even as large as $3a$ along the $[1\bar{1}0]$ direction, the YSR states of the two Fe adatoms hybridize and get split away from the Fermi level. This is only possible because of the long-range nature of the YSR states of Fe hollow-site adatoms (see section S2), which is not observed for YSR states of Fe bridge-site adatoms.

Section 6. As-measured d*I*/d*V*(*V*) and the deconvoluted spectra of each Fe adatom in the Fe-11 chain

Fig. S7A presents differential tunneling conductance (d*I*/d*V*) spectra as a function of energy measured on each Fe hollow-site adatom within the Fe-11 chain, as displayed in Figs. 2E and 2H of the main text. Fe(*n*) denotes the sequentially numbered Fe adatom in the chain, where $n = 1$ is the left chain-end atom. The dashed lines in the panel indicate the positions of the Fermi level (E_F). In Fig. S7B numerically deconvoluted d*I*/d*V* spectra of each Fe adatom within the Fe-11 chain are shown. The dash-dotted lines in the panel indicate the positions of the centers of the YSR bands at ±0.35 meV.

Section 7. Spatial homogeneity of proximity-induced superconductivity in the $BiAg_2$ surface alloy

Fig. S8A shows a constant-current STM image of the bare $BiAg_2$/Ag(111)/Nb(110) sample without any magnetic adatoms on the surface. Besides the presence of a few vacancies of surface Bi atoms, a nanoscale LDOS variation is visible, as discussed in the main text. A spectroscopic $\mathrm{d}I/\mathrm{d}V(V)$ line-profile measured across the bare $BiAg_2$ surface along the $[1\bar{1}0]$ direction is presented in Fig. S8B. The SC coherence peaks observed at ±2.58 meV show no spatial dependence within the accuracy of the STS measurement over a length scale of more than 30 nm, indicating that the proximity-induced superconductivity in the $BiAg_2$ surface alloy is spatially homogeneous and not influenced by the spatial LDOS variations caused by the intrinsic potential disorder of the $BiAg_2$/Ag(111)/Nb(110) heterostructure. The reproducibility of the SC coherence peak positions was analyzed by fitting the individual $\mathrm{d}I/\mathrm{d}V(V)$ curves of the spectroscopic line profile displayed in Fig. S8B with four Lorentzian distribution functions to capture the negative $\mathrm{d}I/\mathrm{d}V$ dips outside the SC coherence peaks as well. An example for the fitting is shown in Fig. S8C. Histograms of the fitted SC coherence peak positions and the peak widths at positive bias voltage, as indicated by the arrow in Fig. S8C, are displayed in Figs. S8D and S8E, respectively. The distribution of the extracted peak widths is centered at around 84 μeV, suggesting that the energy resolution in the STS measurement at the given parameters is on the order of a few tens of μeV. The standard deviation of the extracted peak positions is much smaller than the energy resolution, thereby demonstrating quantitatively the spatial homogeneity of the proximity-induced superconductivity in the $BiAg_2$ layer, despite the presence of nanoscale potential disorder.

Section 8. Comparison of spectral characteristics of Fe-11 chains at various levels of disorder

Fig. S9 shows the spectral characteristics of a few representative Fe-11 chains (along the $[1\bar{1}0]$ direction) constructed atom-by-atom on different locations of the $BiAg_2$/Ag(111)/Nb(110) sample exhibiting various levels of disorder. The dI/dV(eV) line profiles of four different Fe-11 chains (named chain #1 to chain #4) along the chains' center, are presented in Fig. S9, A to D, respectively. We note that all Fe-11 chains have exactly the same structure and $2a$-spacing between the Fe hollow-site adatoms, and they were all built on a defect-free $BiAg_2$/Ag(111) surface region. The electronic structure of the Fe chains, in general, appears to be highly reproducible, in particular regarding the presence of edge states near the Fermi level at both ends and the formation of a YSR band with a band edge around $E - E_F = 0.3$ meV. On the other hand, a random-like fluctuation in the YSR band edge can typically be observed inside the chain. We attribute this effect to residual nanoscale potential disorder due to subsurface defects in the bulk of the Ag(111) islands or at the Ag(111)/Nb(110) interface. However, this type of nanoscale potential disorder does not prevent the emergence of the zero-energy end states which can always be observed, independent of the particular location of the Fe chain relative to the $BiAg_2$/Ag(111)/Nb(110) heterostructure substrate.

Section 9. Ab initio calculations

We prepared a crystal structure based on the $BiAg_2$/Ag(111)/Nb(110) setup as described in the main text. Since the Ag(111) layer is thick, we neglected the Nb(110) substrate. The system we prepared based on the fixed lattice constant of fcc silver, a_{Ag} = 4.0772 Å, is shown in Fig. S10A. There are three substrate layers that are kept fixed, followed by three surface layers that were fully relaxed. Relaxations are performed using a projector augmented wave basis as implemented in VASP[55,56]. We first prepared the $BiAg_2$/Ag(111) system without Fe atoms on the surface. Relaxed Bi atoms are raised by approximately 0.6 Å above the Ag atoms in the $BiAg_2$ surface layer. When we placed Fe atoms in the centers of a Bi triangle, density functional theory (DFT) structure relaxation predicts a significant distortion of the local structure: The side of the Bi triangle shrinks from 4.99 Å to 4.41 Å. Also, the three Bi atoms surrounding the Fe atom are raised further above the Ag surface, by 1.3 Å compared to 0.6 Å for the empty Bi triangular lattice. The substantial structural relaxations around Fe atoms explain why experimentally it seems so difficult to place Fe atoms in linear chains with 1*a* lattice constant because Bi triangle relaxation would be severely hindered. To study the magnetic properties of 2*a*-Fe chains on $BiAg_2$/Ag(111) we prepared the crystal structure shown in Fig. S10B by VASP relaxation for Fe chains. Here, *a* is the Bi-Bi lattice spacing. We then employed DFT calculations based on the full potential local orbital (FPLO) basis set[57] and generalized gradient approximation (GGA) exchange correlation functional[58] to investigate the magnetic interactions of Fe chains on the $BiAg_2$ surface.

For this purpose, we calculated the energies of ferromagnetic (FM) and antiferromagnetic (AFM) states. Note that due to the metallic nature of the Fe chain on $BiAg_2$, the energy mapping is not as precise as for insulating magnets. The bonding between the transition metals and Bi appears relatively strong, however, so that the nature of the bond could have more covalent character than the metallic bonding in the case of Fe on Nb(110)[59]. We found that (i) 2*a*-Fe chains couple ferromagnetically; (ii) the magnetic moments of Fe are $3.1\mu_B$; (iii) the exchange coupling for Fe (unit moments) is about −1.3 meV; (iv) an out-of-plane alignment of the ferromagnetic order is energetically preferred with an anisotropy ΔJ = 5.7 meV.

Section 10. Tight binding calculations: Minimal model and phase diagram

The Hamiltonian studied in the main text is

$$H = H_{\mathrm{surface}} + H_{\mathrm{Fe}} + H_{\Gamma} \tag{S6}$$

with H_{surface} being an effective Hamiltonian for the triangular net of Bi atoms, H_{Fe} the Hamiltonian for the Fe adatoms, and H_{Γ} describing the coupling between Fe adatoms and the Bi surface. This Hamiltonian is in class D, and does not possess mirror symmetry. We approximated the Bi sites with a single orbital to obtain

$$H_{\mathrm{surface}} = \sum_{\langle ij\rangle} t_{ij} c_i^{\dagger} c_j + i\alpha \sum_{\langle ij\rangle} c_{i,\alpha}^{\dagger} (\boldsymbol{\sigma}_{\alpha\beta} \times \boldsymbol{d}_{ij})_z c_{j,\beta} + \Delta_0 \sum_i c_{i,\uparrow}^{\dagger} c_{i,\downarrow}^{\dagger} + \mathrm{H.c.} \tag{S7}$$

where $\langle ij\rangle$ indicates nearest neighbours and i runs over the position of all Bi atoms. We also have $t_{ii} = \mu_i$ being the site-dependent chemical potential, $t_{ij} = 100$ meV the kinetic energy, α the magnitude of conventional Rashba spin-orbit coupling, and Δ_0 the singlet SC order parameter. In the clean limit, $\mu_i = \mu$ is constant for all sites. We chose the lattice vectors $\boldsymbol{a}_1 = a(1/2, \sqrt{3}/2)$ and $\boldsymbol{a}_2 = a(1/2, -\sqrt{3}/2)$. Finally, we have $c_i^{\dagger} = (c_{i,\uparrow}^{\dagger}, c_{i,\downarrow}^{\dagger})$ being the spinor for creating electrons at site i, $\boldsymbol{\sigma}$ the Pauli-matrices, and $\boldsymbol{d}_{ij}$ a unit vector pointing from site j to site i. From the band structure in the normal state given in Ref.[31], with no disorder, we have α = 25 meV and μ = -545 meV (see Fig. 4B of the main text). The remaining terms of the Hamiltonian are:

$$H_{\mathrm{Fe}} = \mu_{\mathrm{Fe}} \sum_r a_r^{\dagger} a_r + J \sum_r a_r^{\dagger} \sigma_z a_r\ , \tag{S8}$$

$$H_{\Gamma} = \Gamma \sum_{\langle ri\rangle} a_r^{\dagger} c_i + H.\mathrm{c}\,. \tag{S9}$$

Here, J is the ferromagnetic Zeeman coupling which represents the magnetic moment of the Fe atoms, μ_{Fe} is the chemical potential of the adatoms, and Γ the hybridization between adatoms and substrate. The index r runs over adatom sites, i.e., the hollow sites of the Bi triangular lattice, and $\langle ri\rangle$ indicates adatom sites r and their adjacent substrate sites i. Furthermore, $a_r^{\dagger} = (a_{r,\uparrow}^{\dagger}, a_{r,\downarrow}^{\dagger})$ is the spinor for electrons on the adatom sites. Additional hopping between the Fe atoms has been omitted as it will be quite small for the $2a$-distance between neighboring Fe atoms. We note, however, that our results change only very little when including this term. In fact, it would further stabilize the topological states and increase the region of the topologically non-trivial phase. Moreover, extending the hoppings in H_{surface} beyond nearest neighbors does not change qualitatively any of our conclusions – as long as the longer-ranged hoppings are significantly weaker than the

nearest-neighbor hoppings. Such weak modifications at best lead to negligible shifts of the boundaries of the topological phase.

Based on the above arguments we chose the following parameters for calculations in the clean limit: (t, α, μ, Δ_0) = (100, 25, -545, 70) meV. To calculate the topological phase diagram, we considered 2a-Fe chains along the $\boldsymbol{a}_1$ direction, with Fe adatoms in the hollow-site positions. To determine the extent of the unit cell in the y-direction (perpendicular to the chain direction), we started with only two Bi atoms and computed the topological phase diagram. Then we repeated this step with four Bi atoms in the y-direction, leading to a similar phase diagram but with shifted phase boundaries. We thus continued increasing the size of the unit cell in the y-direction until we were satisfied that ten Bi atoms in the y-direction suppresses relevant changes of the phase boundaries. Thus, we worked throughout this paper with a unit cell containing 2 ×10 Bi atoms and a single Fe atom. To produce a realistic SC proximity effect, the 20 Bi atoms per unit cell coupled to the SC order parameter being of the order of the SC bulk gap would be insufficient to mimic the 3D bulk of the experimental substrate. To compensate for the relatively small unit cell, we must correspondingly set $\Delta_0 \sim t$ to see the appearance of coherence peaks at the SC bulk edge.

Here, we chose Δ_0 = 70 meV. Assuming strong Fe-substrate coupling, Γ = 35 meV, we found the phase diagram as shown in Fig. 4C of the main text. For weak coupling, the topological phase shrinks and vanishes when Γ < 20 meV. In the phase diagram the topological phase is bounded by two lines, one linear and one a hyperbola, corresponding to gap closings. The linear phase transition is given by $J = \pm(\mu_{\mathrm{Fe}} - 2t)$ while the hyperbola phase transition is given by

$$J \approx \pm\sqrt{\left(\mu_{\mathrm{Fe}} + 2t + \frac{\Gamma^2}{\mu_{\mathrm{Fe}} - 2t}\right)^2 + \Gamma^2 {\Delta_0}^2 (\mu_{\mathrm{Fe}} - 2t)^2} \qquad \text{(S10)}$$

To see coherence peaks at the SC bulk edge for calculations with open boundary conditions, we require many sites in at least one crystalline direction. This corresponds to a finely discretized Brillouin zone, or in other words, yields many states around the bulk edge. As discussed for the periodic Hamiltonian, ten sites in width is sufficient. A wider substrate could be simulated, but this will greatly increase the computational cost of the disorder runs with little benefit.

Section 11. Analysis of correlated disorder

In contrast to the clean limit, we considered now a site-dependent μ_i which combines the chemical potential μ and non-magnetic random disorder $\delta\mu_i$, $\mu_i = \mu + \delta\mu_i$. For the disorder analysis of our effective substrate of Bi atoms, we chose the following parameters: (t, α, μ, J, μ_{Fe}, Δ_0) = (100, 25, -545, 22, 30, 70) meV. We modeled the disordered $BiAg_2$ surface by applying a correlated potential disorder $\delta\mu_i$ to the substrate sites. We drew values from a Gaussian noise source N_G with mean $\mu = -545$ meV and standard deviation D. We applied a low-pass filter to this noise source via

$$N = F_r^{-1}\left[F_k[\delta\mu_i]e^{-k^2/K_c{}^2}\right] \quad \text{(S11)}$$

with $F_{r/k}$ the Fourier transform over $\boldsymbol{r}$ or $\boldsymbol{k}$, respectively, and K_c the absolute value of the cutoff wavevector. This low-pass filter eliminates fluctuations in the disorder potential with wavenumber $k > K_c$. We chose K_c = (0.25, 0.01), corresponding to nanoscale correlations along the $\boldsymbol{a}_1$ direction. We show in Fig. 2L of the main text an example of the chemical potentials μ_i produced by this method, resolved in real space. We also plot the complementary histogram of chemical potentials, and the Gaussian noise source (see Fig. 2L of the main text). We simulated several choices of D and find that Majorana modes are robust up to $D \gg \Delta$ for potential disorder on substrate sites.

We note that other types of disorder lead to comparable results to those given in the main text. To demonstrate this, we first considered correlated disordered hoppings ("bond disorder") between the substrate atoms. We followed the same strategy to correlate the disorder as explained above. In Fig. S11 we show plots analogous to the main text but for disordered substrate hoppings. Also for this type of disorder we can close the bulk gap with increasing disorder strength D. For $D \approx 50$ meV the bulk gap cannot longer be resolved (see DOS plot at first site in Fig. S11A), but well-pronounced zero-energy peaks are still visible. As in the main text, the DOS plot is disorder-averaged over 100 disorder realizations; the blue curve is the average, and the faint blue region corresponds to twice the standard deviation. In Fig. S11B we show the LDOS as a function of energy E and position x (integrated over y) for a single representative disorder realization. For $E = 0$, we also show in the lower panel the LDOS(x, y) with well-defined spectral weight at the chain ends. Both plots show some aperiodic behavior, as expected from the random disorder and in line with the experimental observation. As for the previous case, we found that Majorana modes are robust up to $D \gg \Delta$.

As a second case, we considered disorder in the hybridization (i.e., the hoppings) between the magnetic adatoms and the substrate atoms. As the chain is only 11 sites long, and each adatom is coupled to its three neighboring Bi atoms, only a total of 33 hoppings is affected. Thus we did not correlate the disorder for this example, but do not expect any significant difference if we did. The effect of disorder in the substrate is clearly much weaker for the topological chain, compared to disorder which couples directly to the chain as is the case here. In line with this expectation, we found that the topological gap is already closed for $D \approx 10$ meV; as shown in Fig. S11C, well-pronounced peaks at $E = 0$ can still be observed in the DOS of the first site. In Fig. S11D we show again the LDOS for a single representative disorder realization, substantiating that the gap is closed, and that the $E = 0$ peaks correspond to localized states at the chain ends. Majorana modes remain robust for even larger values of D but in contrast to the other considered types of disorder, the topological phase breaks down when $D \gg \Delta$. As observed for the other disorder types, the aperiodicity within the chain is well pronounced.

We expect that other possible types of disorder such as potential disorder on the chain and disordered hoppings between adatoms would lead to comparable results. It is obvious that the specific type of disorder is not essential – what matters is only the presence of non-magnetic disorder. The specific type of disorder only rescales the critical disorder strength D_c where topological superconductivity breaks down. These findings let us to conclude that in the topological phase we see the zero-bias peak due to Majorana modes to be robust to disorder, even when the topological gap vanishes.

References

40. Franke K. J., Schulze G., and Pascual J. I. Competition of Superconducting Phenomena and Kondo Screening at the Nanoscale. *Science* **332**, 940 (2011).

41. Bardeen J., Cooper L. N., and Schrieffer J. R. Theory of Superconductivity. *Phys. Rev.* **108**, 1175 (1957).

42. Dynes R. C., Narayanamurti V., and Garno J. P. Direct Measurement of Quasiparticle-Lifetime Broadening in a Strong-Coupled Superconductor. *Phys. Rev. Lett.* **41**, 1509 (1978).

43. Twomey S. On the Numerical Solution of Fredholm Integral Equations of the First Kind by the Inversion of the Linear System Produced by Quadrature. *J. Assoc. Comput. Mach.* **10**, 97 (1963).

44. Ternes M., Schneider W.-D., Cuevas J.-C., Lutz C. P., Hirjibehedin C. F., and Heinrich A. J. Subgap structure in asymmetric superconducting tunnel junctions. *Phys. Rev. B* **74**, 132501 (2006).

45. Yu L. Bound state in superconductors with paramagnetic impurities. *Acta Phys. Sin.* **21**, 75 (1965).

46. Shiba H. Classical Spins in Superconductors. *Prog. Theor. Phys.* **40**, 435 (1968).

47. Rusinov A. I. Superconductivity near a Paramagnetic Impurity. *JETP Lett.* **9**, 85 (1969).

48. Fetter A. L. Spherical Impurity in an Infinite Superconductor. *Phys. Rev.* **140**, A1921 (1965).

49. Yazdani A., Jones B. A., Lutz C. P., Crommie M. F., and Eigler D. M., Probing the Local Effects of Magnetic Impurities on Superconductivity. *Science* **275**, 1767 (1997).

50. Ménard G. C., Guissart S., Brun C., Pons S., Stolyarov V. S., Debontridder F., Leclerc M. V., Janod E., Cario L., Roditchev D., Simon P., and Cren T. Coherent long-range magnetic bound states in a superconductor. *Nature Phys.* **11**, 1013 (2015).

51. Kim H., Rózsa L., Schreyer D., Simon E., and Wiesendanger R. Long-range focusing of magnetic bound states in superconducting lanthanum. *Nature Commun.* **11**, 4573 (2020).

52. Rütten L. M., Liebhaber E., Rossnagel K., Franke K. J. Charge-Density-Wave Control by Adatom Manipulation and Its Effect on Magnetic Nanostructures. *Nano Lett.* **25**, 115 (2025).

53. Wiesendanger R. Spin mapping at the nanoscale and atomic scale. *Rev. Mod. Phys.* **81**, 1495 (2009).

54. Schneider L., Beck Ph., Rozsa L., Posske Th., Wiebe J. and Wiesendanger R. Probing the topologically trivial nature of end states in antiferromagnetic atomic chains on superconductors. *Nature Commun.* **14**, 2742 (2023).

55. Kresse G. and Hafner J. Ab initio molecular dynamics for liquid metals. *Phys. Rev. B* **47**, 558 (1993).

56. Kresse G. and Furthmüller J. Efficiency of ab-initio total energy calculations for metals and semiconductors using a plane-wave basis set. *Comp. Mater. Sci.* **6**, 15 (1996).

57. Koepernik K. and Eschrig H. Full-potential nonorthogonal local-orbital minimum-basis band-structure scheme. *Phys. Rev. B* **59**, 1743 (1999).

58. Perdew J. P., Burke K., and Ernzerhof M. Generalized Gradient Approximation Made Simple. *Phys. Rev. Lett.* **77**, 3865 (1996).

59. Crawford D., Mascot E., Shimizu M., Wiesendanger R., Morr D. K., Jeschke H. O., and Rachel S. Increased localization of Majorana modes in antiferromagnetic chains on superconductors. *Phys. Rev. B* **107**, 075410 (2023).

Figure S1.

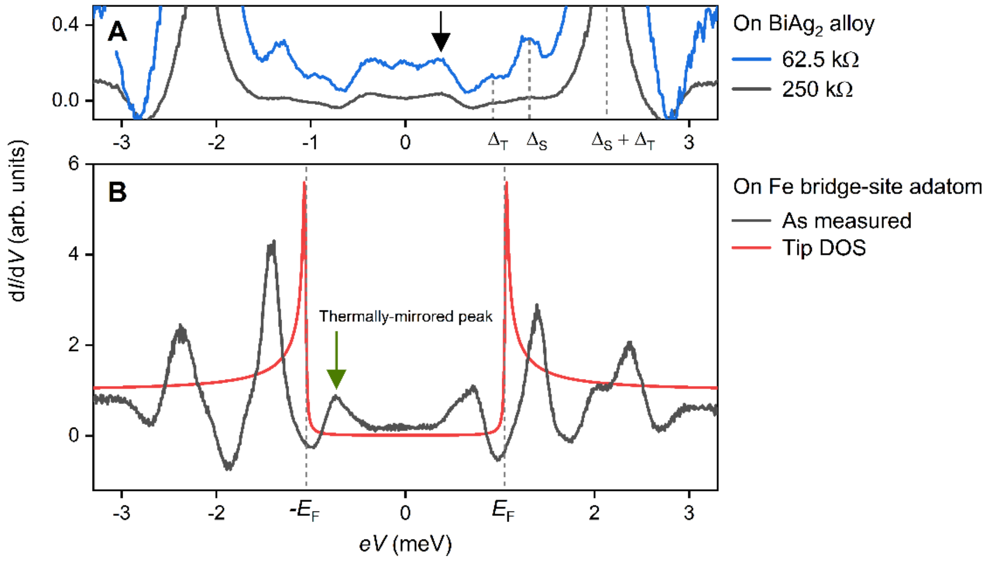

Fig. S1. Numerical deconvolution of tunneling spectra from Superconductor-Insulator-Superconductor (SIS) junctions. (**A**) Differential tunneling conductance (d*I*/d*V*) spectrum as a function of energy (*eV*) measured on the $BiAg_2$ surface alloy at two close tip-sample distances, where the closer one is corresponding to a lower junction resistance. Dashed lines indicate the determined superconducting (SC) energy gaps of the Nb-tip Δ_T and the sample surface Δ_S as well as their sum $\Delta_S + \Delta_T$. (**B**) An exemplary d*I*/d*V*(*eV*) spectrum measured on top of an Fe bridge-site adatom. The determined Fermi level (E_F) of the sample surface is shown as dashed lines. The Dynes density of states (DOS) of the tip with a finite quasiparticle lifetime Γ, which is used for the numerical deconvolution, is displayed as well (see equation S2). The parameters to describe the tip's DOS are $\Delta_T = 1.05$ meV and Γ = 16 μeV. The arrows in both panels indicate the mirrored peaks caused by thermal excitation. Measurement parameters: $T = 4.2$ K, $V_{stab} = 5$ mV, $V_{mod} = 40$ μV, $f_{mod} = 1.2$ kHz. For (**B**): $I_{stab} = 1$ nA.

Figure S2.

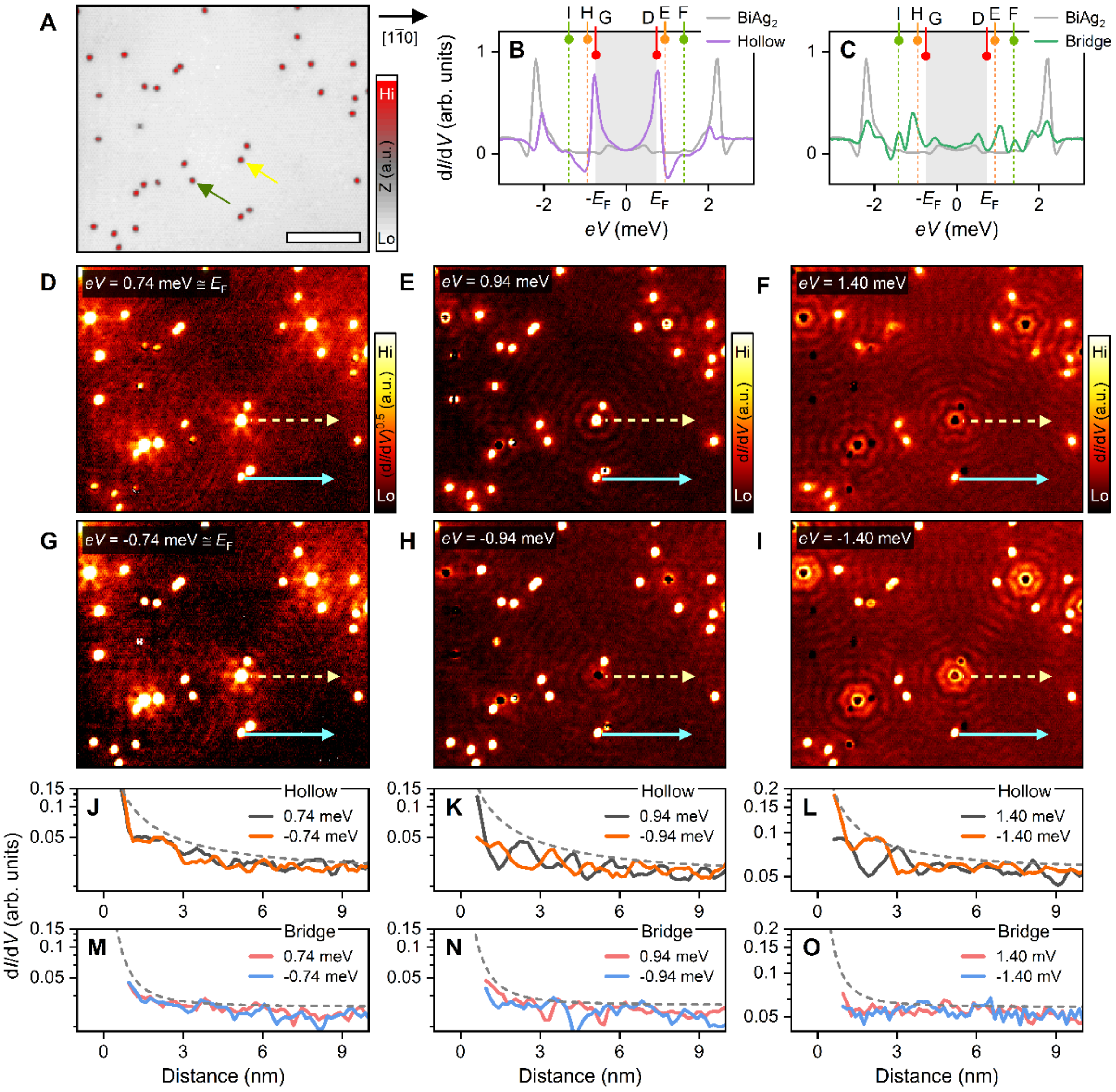

Fig. S2: Long-range nature of YSR states of single Fe adatoms on the $BiAg_2$ surface alloy. (**A**) Constant-current STM image of the $BiAg_2$ surface alloy with Fe bridge-site (green arrow) and Fe hollow-site (yellow arrow) adatoms. The scale bar corresponds to 10 nm. (**B**) Differential tunneling conductance (dI/dV) spectra as a function of energy measured on Fe hollow-site and (**C**) bridge-site adatoms on $BiAg_2$. The vertical arrows and dashed lines in (**B**) and (**C**) indicate the energies for which spectroscopic maps were obtained, corresponding to panels (**D**) to (**I**). The dI/dV spectrum measured on the bare $BiAg_2$ surface alloy is additionally plotted for comparison.

The shaded areas indicate the positions of the Fermi level (E_F). (**D** to **I**) Spectroscopic dI/dV maps for the area presented in panel (**A**) at representative energies inside the superconducting gap (see arrows in panels (**B**) and (**C**)). (**J** to **L**) Line cuts of the dI/dV maps near the Fe hollow-site adatom along the $[1\bar{1}0]$ direction, as indicated by dashed arrows in panels (**D**) to (**I**), on a semi-logarithmic scale. (**M** to **O**) Line cuts of the dI/dV maps near the Fe bridge-site adatom, as indicated by solid arrows in panels (**D**) to (**I**). Dashed lines in panels (**J**) to (**O**) are guided envelope functions (see text). Measurement parameters: for all; T = 4.2 K, V_{stab} = 5 mV, I_{stab} = 0.5 nA, f_{mod} = 1.097 kHz. For (**B**) and (**C**): V_{mod} = 50 μV, and for all other measurements: V_{mod} = 0.1 mV.

Figure S3.

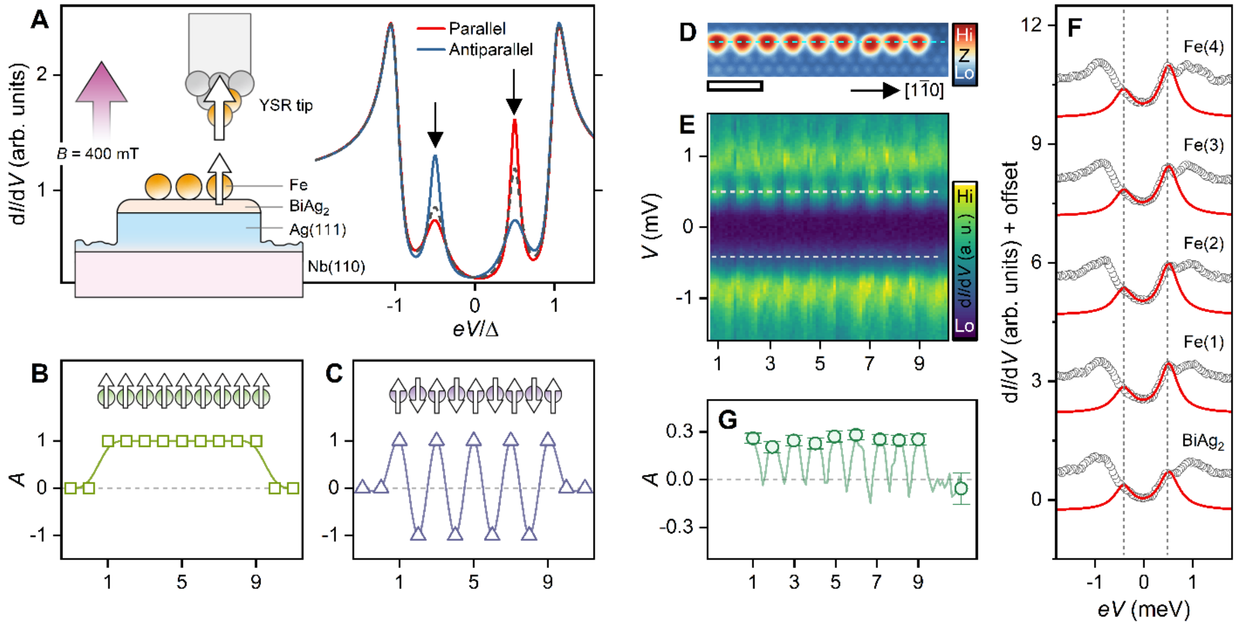

Fig. S3. Ferromagnetic state of the bottom-up fabricated 2a-Fe chains on the $BiAg_2$ surface alloy. (**A**) Scheme of spin-polarized scanning tunneling spectroscopy (SP-STS) using a YSR-tip applied to bottom-up constructed Fe chains on a $BiAg_2$ surface alloy prepared on Ag(111)/Nb(110). (**B** to **C**) Schematics of the expected line profile of the spin asymmetry A extracted from SP-STS measurements on (**B**) a ferromagnetic 9-atom chain and (**C**) an antiferromagnetic 9-atom chain. The dashed lines represent the expected value of zero for A in case of a non-magnetic system. (**D**) Constant-current STM image of an artificially constructed Fe chain on $BiAg_2$/Ag(111)/Nb(110). The scale bar corresponds to 2 nm. (**E**) Line profile of the bias-dependent differential tunneling conductance (dI/dV) measured along the center of the Fe chain, as indicated by the horizontal dashed line in panel (D). The x-axis denotes the sequentially numbered Fe atoms from the left to the right side of the chain. (**F**) Individual dI/dV spectra measured above the four Fe adatoms starting from the left end of the chain together with a dI/dV spectrum of the bare $BiAg_2$/Ag(111)/Nb(110) substrate. The solid red lines are fits to the measured spectroscopic data from which the values of the spin asymmetry A have been extracted. The dashed lines indicate the positions of the YSR peaks. (**G**) Line profile of the spin asymmetry A extracted from the SP-STS data of the Fe chain revealing the presence of ferromagnetic order within the chain. The open symbols represent the determined values of A at each Fe adatom position and for the bare $BiAg_2$

surface (right-hand side). The error bars are the 95 % confidence values obtained from the fits near the YSR peaks, shown as solid lines in panel (F). Measurement parameters: T = 0.32 K, V_{stab} = 5 mV, I_{stab} = 0.5 nA; for (**E**) and (**F**): B_{ext} = 0.4 T, V_{mod} = 0.1 mV, f_{mod} = 4.55 kHz.

Figure S4.

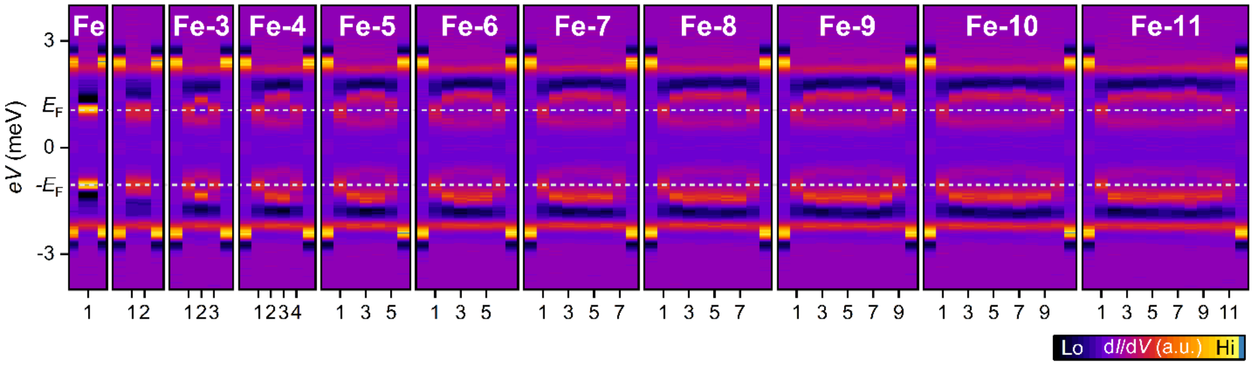

Fig. S4. Evolution of the spectral characteristics of 2a-Fe chains as a function of chain length. Line profiles of the differential tunneling conductance (d*I*/d*V*) measured along the center of Fe-*n* chains from Fe-1 (left) to Fe-11 (right), where *n* is the number of Fe adatoms in the chains with an interatomic distance of 2*a*. The horizontal dashed lines correspond to the Fermi level (E_F) of the sample. Measurement parameters: T = 4.2 K, I_{stab} = 1 nA, V_{stab} = 5 mV, f_{mod} = 1.234 kHz, and V_{mod} = 40 μV.

Figure S5.

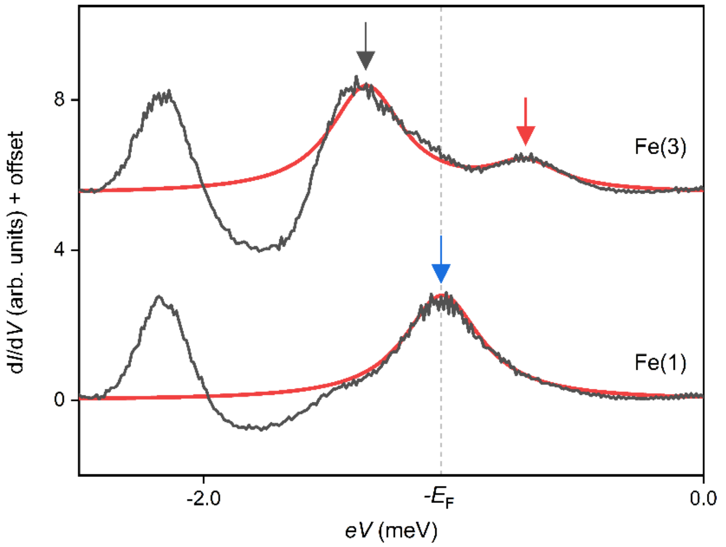

Fig. S5: Analysis of the line-width of the zero-energy end state vs. the width of the YSR bulk band of the Fe-11 chain. Differential tunneling conductance (d*I*/d*V*) spectra as a function of energy for negative sample bias measured above the end atom Fe(1) and bulk atom Fe(3) positions of the Fe-11 chain, as shown in Fig. 2J of the main text. The red solid lines are the fitted curves for the peaks near the Fermi level E_F (dashed line), where a single or two Lorentzian functions were used to determine the positions and line-widths of the states for the end and bulk atom, respectively. The vertical arrows indicate the determined peak positions of the end mode at zero energy (blue arrow), the center of the bulk band at about $E–E_F = –0.35$ meV (black arrow), and its thermally mirrored peak (red arrow). The line-widths of the zero-energy mode as well as the YSR bulk band are about 0.2 meV. The spectra are offset for clarity.

Figure S6.

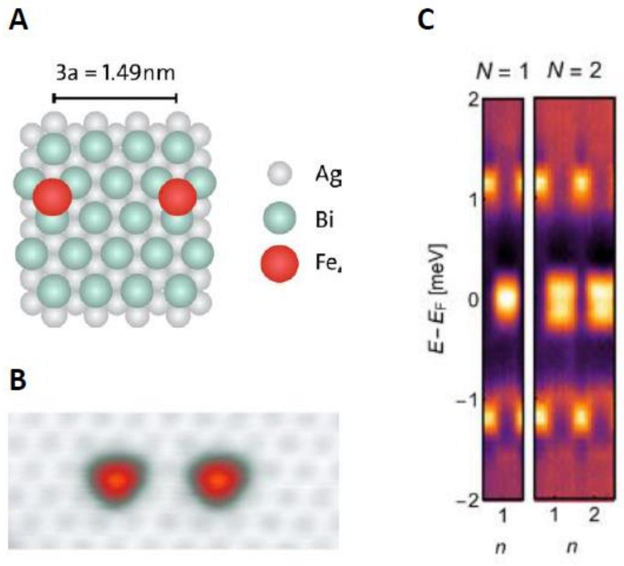

Fig. S6. Hybridization induced splitting of the YSR state for an Fe pair. (**A**) Schematic of a pair of Fe hollow-site adatoms with a distance of 3*a* on the $BiAg_2$ surface alloy. (**B**) Constant-current STM image of such Fe pair. (**C**) Line profiles of the deconvoluted differential tunneling conductance (d*I*/d*V*) spectra obtained for an individual Fe hollow-site adatom and along the center of a pair of Fe hollow-site adatoms with a distance of 3*a*. The hybridization-induced splitting of the YSR state upon formation of the Fe pair is clearly visible even for an Fe-Fe distance of 1.49 nm. Measurement parameters: T = 4.2 K, I_{stab} = 1 nA, V_{stab} = -5 mV, and V_{mod} = 50 μV.

Figure S7.

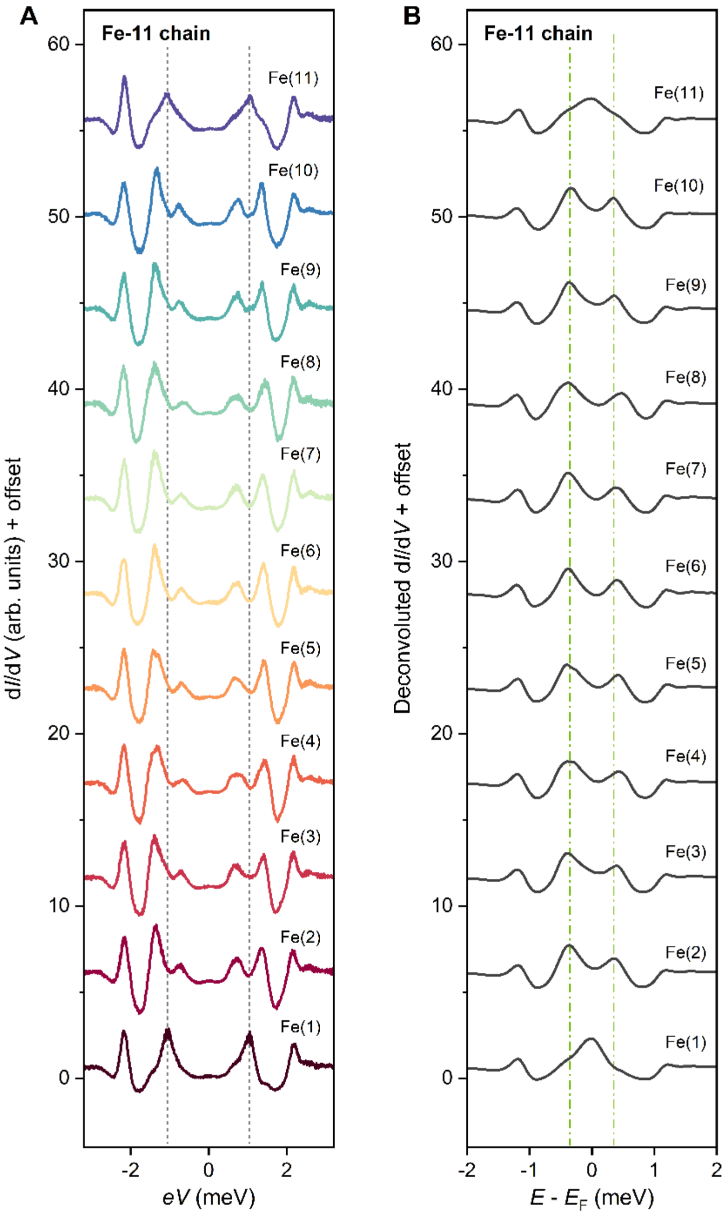

Fig. S7: As-measured d*I*/d*V*(*eV*) and the deconvoluted spectra of each Fe adatom in the Fe-11 chain. (**A**) Differential tunneling conductance spectra (d*I*/d*V*) as a function of energy as measured on each Fe hollow-site adatom within the Fe-11 chain, displayed in Figs. 2E and 2H of the main text. Fe(*n*) denotes the sequentially numbered Fe adatom in the chain, where $n = 1$ is the left chain-end atom. The dashed lines indicate the positions of the Fermi level (E_F). (**B**) Numerically deconvoluted d*I*/d*V* spectra of each Fe adatom within the Fe-11 chain. The dash-dotted lines in the

panel indicate the centers of the YSR bands at ±0.35 meV. All the spectra are offset for clarity. Measurement parameters: T = 4.2 K, I_{stab} = 1 nA, V_{stab} = 5 mV, V_{mod} = 40 μV.

Figure S8.

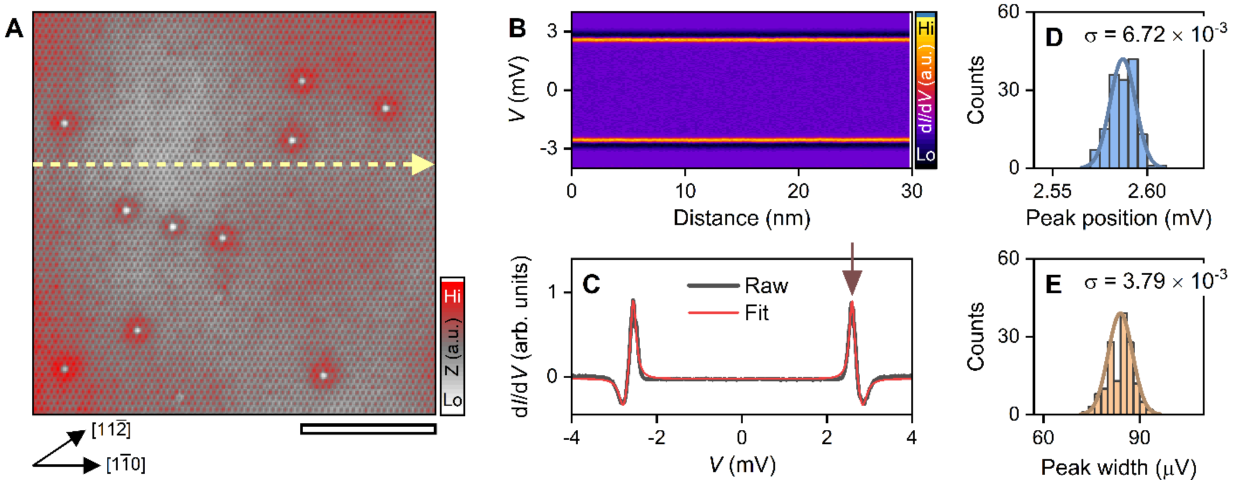

Fig. S8. Spatial homogeneity of proximity-induced superconductivity in the $BiAg_2$ surface alloy. (**A**) Constant-current STM image of the $BiAg_2$ surface alloy without Fe adatoms. The scale bar corresponds to 10 nm. (**B**) Line profile of the differential tunneling conductance as a function of sample bias, dI/dV(V), along the $BiAg_2$ surface as indicated by the dashed arrow in panel (**A**). The spatial homogeneity of the proximity-induced superconducting (SC) state is apparent despite the nanoscale LDOS variations observed in (**A**). (**C**) Representative dI/dV(V) spectrum selected from panel (**B**), plotted with the best-fit curve using four Lorentzian functions. Measurement parameters: T = 320 mK, I_{stab} = 1 nA, f_{mod} = 4.142 kHz, V_{mod} = 20 μV; for (**A**): V_{stab} = 50 mV; for (**B**) and (**C**); V_{stab} = 6 mV. (**D** to **E**) Histogram of (**D**) the SC coherence peak positions and (**E**) peak widths extracted from the spectroscopic line profile in (**B**) using the fitting procedure at positive sample bias, as indicated by vertical arrow in panel (**C**), along with the Gaussian distribution curves (solid lines). The values for the standard deviation of both distributions are given at the top of each panel.

Figure S9.

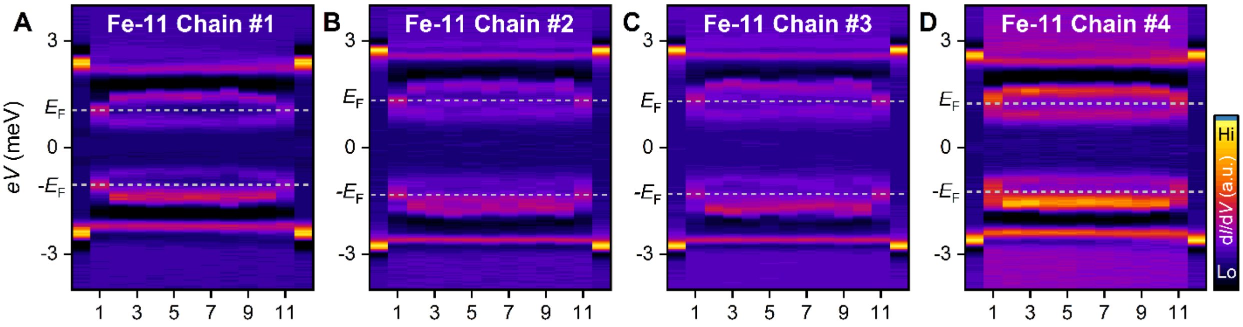

Fig. S9. Comparison of spectral characteristics of Fe-11 chains at various levels of disorder. (**A** to **D**) Line profiles of energy-dependent differential tunneling conductance (d*I*/d*V*) of a few representative Fe-11 chains with 2*a* spacing for various levels of potential disorder. The spectra were measured along the center of Fe-11 chains which were bottom-up fabricated on different $BiAg_2$/Ag(111) islands on Nb(110). The horizontal dashed lines correspond to the Fermi level (E_F) of the sample. The *x*-axes denote the sequentially numbered Fe atom from the left to the right side of the chains. Measurement parameters: T = 4.2 K, V_{stab} = 5 mV, and V_{mod} = 40 µV; for (**A**): I_{stab} = 1 nA, f_{mod} = 1.234 kHz; for (**B**): I_{stab} = 0.4 nA, f_{mod} = 4.55 kHz; for (**C**): I_{stab} = 0.4 nA, f_{mod} = 2.22 kHz, and for (**D**): I_{stab} = 0.5 nA, f_{mod} = 2.22 kHz.

Figure S10.

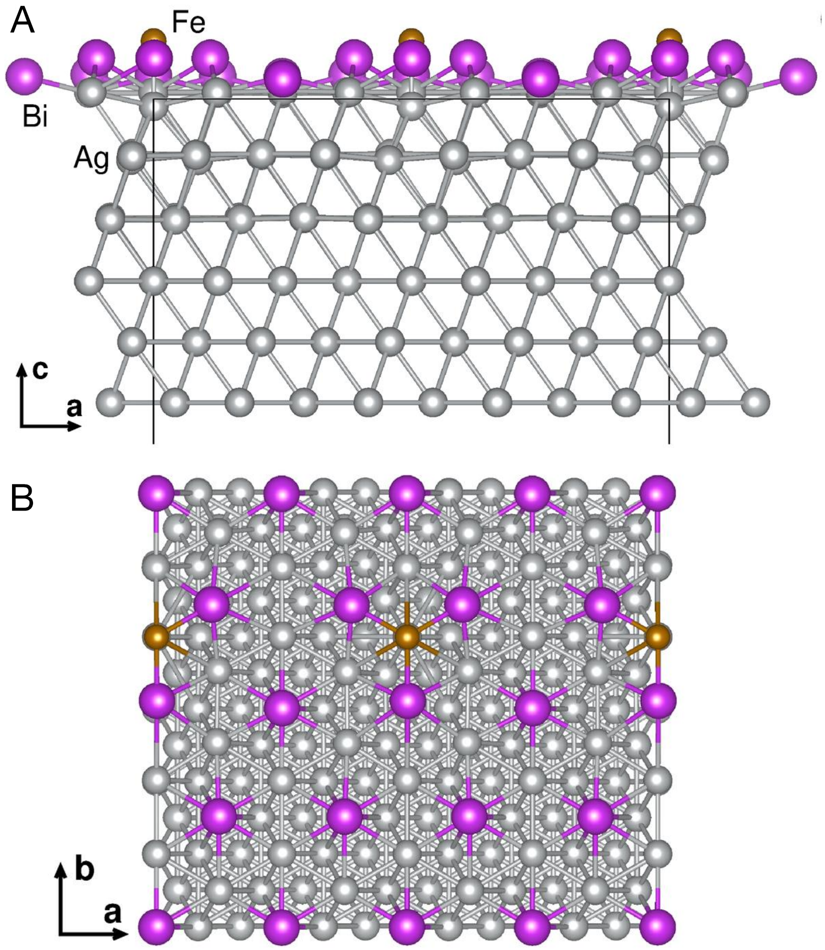

Fig. S10. Ab initio calculations. (**A**) View of the *a*-*c* plane of the $BiAg_2$/Ag(111) heterostructure (side view) revealing six Ag(111) layers: the three bottom layers are fixed, while the top three layers are relaxed. The top layer forms the $BiAg_2$ alloy. (**B**) Top view showing the relaxation of the Bi atoms surrounding Fe atoms (Ag = silver, Bi = purple, Fe = bronze). Fe atoms are separated by a distance of 2*a*.

Figure S11.

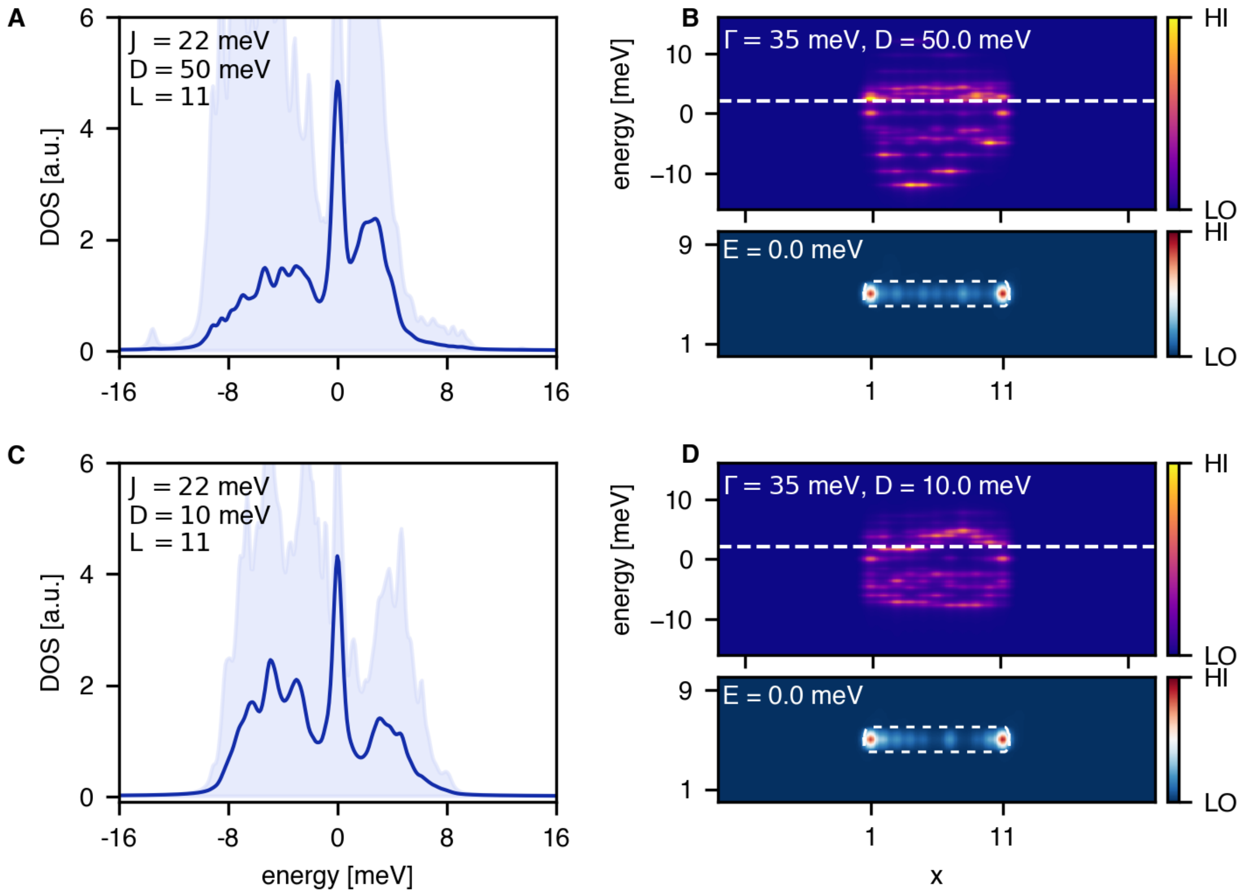

Fig. S11. Theoretical analysis of the influence of other types of disorder on Majorana zero modes. (**A**) Averaged density of states (DOS) at the ends of Fe chains in the topological phase with $L = 11$ for correlated bond disorder between substrate atoms. The solid lines indicate the mean and the shaded regions twice the standard deviation for 100 disorder realizations. (**B**) Local density of states LDOS(E, x) plots for one disorder realization in the topological phase for chain length $L = 11$ for correlated bond disorder between substrate atoms. (**C**), (**D**) Same as **A** and **B**, but for bond disorder between magnetic adatoms and their neighboring substrate atoms. For all plots we used $\mu_{Fe} = 30$ meV and $J = 22$ meV.
